# CDK4/6 inhibition blocks cancer metastasis through a USP51-ZEB1-dependent deubiquitination mechanism

**DOI:** 10.1038/s41392-020-0118-x

**Published:** 2020-03-11

**Authors:** Zhen Zhang, Jianjun Li, Yang Ou, Guang Yang, Kaiyuan Deng, Qiong Wang, Zhaoyang Wang, Wenhao Wang, Quansheng Zhang, Hang Wang, Wei Sun, Peiqing Sun, Shuang Yang

**Affiliations:** 1 Tianjin Key Laboratory of Tumor Microenvironment and Neurovascular Regulation, Medical College of Nankai University, Tianjin, 300071 China; 2 College of Pharmacy, Nankai University, Tianjin, 300071 China; 3 Tianjin Key Laboratory of Organ Transplantation, Tianjin First Center Hospital, Tianjin, 300192 China; 4 Department of Cancer Biology, Wake Forest University School of Medicine, Winston-Salem, NC 27157 USA

**Keywords:** Breast cancer, Epigenetics

## Abstract

Tumor metastasis is the most common cause of cancer-related deaths, yet it remains poorly understood. The transcription factor zinc-finger E-box binding homeobox 1 (ZEB1) is involved in the epithelial-to-mesenchymal transition (EMT) and plays a pivotal role in tumor metastasis. However, the underlying mechanisms of the posttranslational modification of ZEB1 remain largely unknown. Herein, we demonstrated that specific inhibition of CDK4/6 was able to block tumor metastasis of breast cancer by destabilizing the ZEB1 protein in vitro and in vivo. Mechanistically, we determined that the deubiquitinase USP51 is a bona fide target of CDK4/6. The phosphorylation and activation of USP51 by CDK4/6 is necessary to deubiquitinate and stabilize ZEB1. Moreover, we found a strong positive correlation between the expression of *p*-RB (an indicator of CDK4/6 activity), *p*-USP51 and ZEB1 in metastatic human breast cancer samples. Notably, the high expression of *p*-RB, *p*-USP51, and ZEB1 was significantly correlated with a poor clinical outcome. Taken together, our results provide evidence that the CDK4/6-USP51-ZEB1 axis plays a key role in breast cancer metastasis and could be a viable therapeutic target for the treatment of advanced human cancers.

## Introduction

Tumor metastasis is responsible for the majority of cancer-associated mortalities.^1,2^ It is well established that tumor metastasis results from cancer cells that have left the primary tumor mass and traveled through the body’s highways—the blood and lymphatic vessels—to new sites throughout the body where they can establish new colonies.^3^ Many studies suggest that zinc-finger E-box binding homeobox 1 (ZEB1), acting as a key transcription factor of EMT, plays an important role in tumor metastasis by facilitating cell migration and invasion.^4,5^ ZEB1 is a member of the zinc-finger homeodomain transcription factors, which contains two widely separated zinc-finger clusters that are located towards the N- and C-terminal ends of the protein.^6^ These zinc-finger clusters enable ZEB1 to bind to specific DNA sequences [CA(C/G)(C/G)TG], which are collectively called the E_2_-box of its target genes (e.g., CDH1, the gene encoding E-cadherin), to repress gene expression while promoting EMT and cancer metastasis.^6–8^

Notably, various studies have shown that the expression of ZEB1 is regulated by multiple signaling pathways at the transcriptional level, such as transforming growth factor-β (TGF-β), Wnt and Notch.^9,10^ For example, the p65 subunit of NF-κB can activate ZEB1 transcription by directly binding to its promoter, which leads to an EMT phenotype in MCF-10A breast epithelial cells.^11^ Another recent report by Jin et al. demonstrated that the ubiquitin-conjugating enzyme E2 C (UBE2C) functions as an oncogene that promotes EMT in non-small-cell lung cancer (NSCLC) by directly targeting the 5′-UTR of the transcript encoding ZEB1.^12^ In addition, the expression of ZEB1 is tightly regulated by a variety of microRNAs at the posttranscriptional level.^13–15^ It has been well established that miR-200 family members (e.g., miR-141, miR-200a and miR-200c) facilitate the degradation of ZEB1 mRNA by directly binding to its 3′-UTR, which results in distinct tumor phenotypes such as stemness and distant metastasis.^15–17^ However, only a few studies have focused on the mechanisms of the posttranslational modification of ZEB1. Chen et al. previously reported that the ubiquitin ligase Siah decreased the stability of the ZEB1 protein through the ubiquitin-proteasome pathway, which subsequently affects the EMT process in mammalian cancer cells.^18^ Moreover, hyperactivation of the ataxia telangiectasia mutated (ATM) kinase has been shown to directly phosphorylate and stabilize ZEB1 in response to DNA damage in radioresistant breast cancer cells.^19^ Thus, further elucidating the underlying mechanisms of ZEB1 posttranslational regulation will identify new therapeutic strategies to deplete ZEB1 expression and to overcome metastasis and therapy resistance in human cancers.

Deubiquitinating enzymes (DUBs) are key components of the ubiquitin-proteasome system (UPS) that remove ubiquitin chains from their protein substrates.^20^ A large body of evidence suggests that the dysfunction of DUBs is responsible for a multitude of pathologies, including cancer.^21,22^ More recently, there have been reports of associations between DUBs and metastasis in various cancer types. For example, the ectopic expression of USP14 is associated with liver and lymph node metastasis in colorectal cancer.^23^ Multiple deubiquitinating enzymes, including USP26, OTUB1 and PMSD3, have been shown to promote the metastasis of esophageal squamous cell carcinoma through the stabilization of Snail, which is another EMT transcription factor.^24–26^ Notably, WP1130, which is a partially selective inhibitor of several DUBs, including USP9X, USP5, USP14, and UCH37, has been shown to trigger a rapid accumulation of polyubiquitinated proteins into aggresomes and induce breast tumor regression,^27^ which suggests a potential role for DUBs as therapeutic targets in the treatment of cancer metastasis.

In the present study, we provide evidence that the specific inhibition of CDK4/6 results in a significant decrease in ZEB1 protein stability that subsequently blocks tumor metastasis in breast cancer both in vitro and in vivo. Moreover, the deubiquitinase USP51 was identified as a bona fide target of CDK4/6. At the molecular level, CDK4/6 phosphorylates and activates USP51, which is then responsible for the deubiquitination and stabilization of ZEB1. Importantly, we also demonstrated a strong positive correlation between the expression of *p*-RB (an indicator of CDK4/6 activity), *p*-USP51 and ZEB1 in human breast tumor samples. Collectively, our data show that the CDK4/6-USP51-ZEB1 axis might play a key role in breast cancer metastasis, which can serve as a basis for the future development of therapeutic interventions in the treatment of advanced cancers.

## Results

### CDK4/6 inhibition induces ZEB1 protein degradation

We used Western blotting to screen an in-house library of natural compounds to identify those that could potentially alter ZEB1 protein stability (Supplementary Table S1) through their influence on ZEB1 expression in MDA-MB-231 breast cancer cells. An isoflavone compound, biochanin A, was shown to significantly downregulate ZEB1 protein levels (Supplementary Table S1 and Supplementary Fig. S1a). Furthermore, a dose-dependent inhibition of ZEB1 protein levels was confirmed in MDA-MB-231 and SUM-159 breast cancer cells in response to treatment with 50–150 μM biochanin A (Supplementary Fig. S1b). However, the quantitative PCR results showed that the changes in ZEB1 mRNA levels were not as evident (Supplementary Fig. S1c), which demonstrates that biochanin A predominantly regulates ZEB1 protein stability.

Consequently, we treated MDA-MB-231 and SUM-159 cells with the protein synthesis inhibitor cycloheximide (CHX) in the presence or absence of biochanin A. We showed that a combined treatment with biochanin A and CHX led to a rapid decrease in ZEB1 protein levels at the indicated time points (Supplementary Fig. S1d), which implies that biochanin A might promote ZEB1 protein degradation. The cells were further treated with either the proteasomal inhibitor MG132 or the lysosomal inhibitor chloroquine (CQ) in the presence or absence of biochanin A. We found that the biochanin A-induced downregulation of ZEB1 protein was significantly attenuated by MG132 addition (Supplementary Fig. S1e); however, CQ had no effect (Supplementary Fig. S1f). In line with this, the ubiquitination assay further showed that treatment with biochanin A increased the polyubiquitination levels of ZEB1 protein in the presence of MG132 (Supplementary Fig. S1g), confirming a role for biochanin A in the induction of ZEB1 protein degradation via the ubiquitin-proteasome pathway.

To elucidate the potential mechanism that mediates the ubiquitination-dependent degradation of ZEB1 by biochanin A, we performed a computational molecular dynamics simulation and found that the CDK4 and CDK6 kinases had strong affinities for biochanin A (Supplementary Fig. S1h). Moreover, the cellular thermal shift assay demonstrated that treatment with biochanin A efficiently stabilized CDK4/6 in MDA-MB-231 cells, indicating that biochanin A targets CDK4/6 directly. We also used palbociclib, a highly selective CDK4/6 inhibitor, to confirm the results (Supplementary Fig. S1i), and our findings collectively suggest that biochanin A might induce ZEB1 protein degradation by targeting CDK4/6. Notably, MDA-MB-231 and SUM-159 cells were synchronized in the G1/S phase using methotrexate and in the G2/M phase using colchicine, respectively.^28^ In accordance with the high CDK4/6 activity during the G1/S phase,^29,30^ we observed significantly increased expression of ZEB1 protein (Supplementary Fig. S1j). However, the protein level of ZEB1 was downregulated during the M phase along with decreased CDK4/6 activity.

**Fig. 1:**
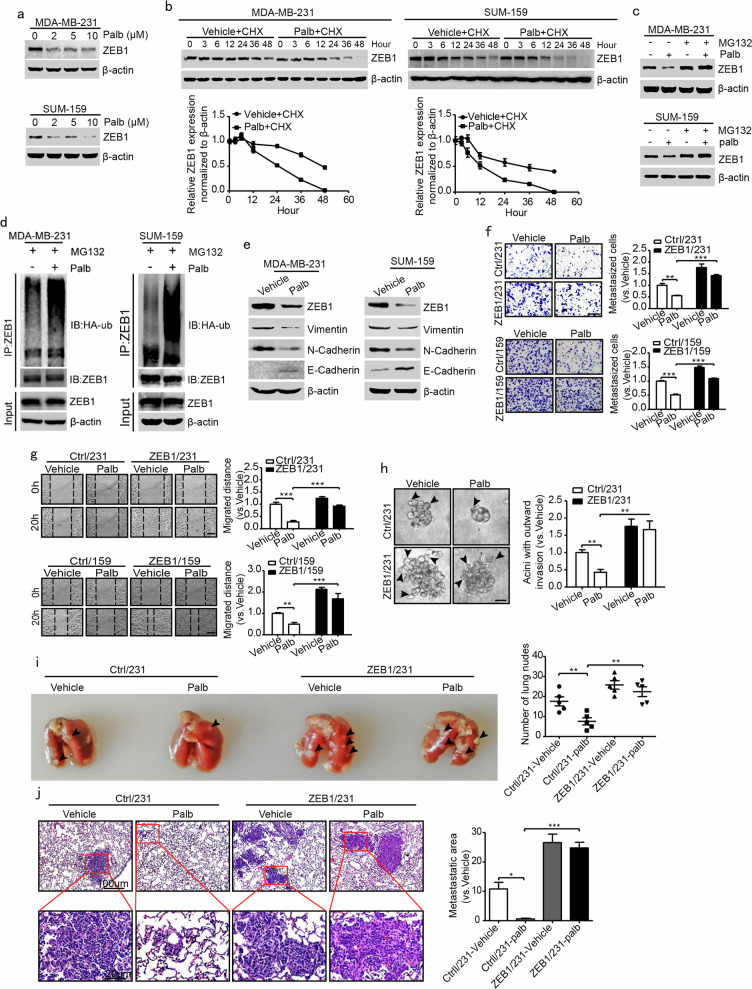
CDK4/6 inhibition blocks breast cancer metastasis by inducing ZEB1 protein degradation. **a** Western blotting of ZEB1 expression in MDA-MB-231 and SUM-159 cells after treatment with the indicated concentrations of palbociclib for 48 h. **b** CHX pulse-chase analysis of ZEB1 protein stability in MDA-MB-231 and SUM-159 cells after treatment with palbociclib at the indicated time points. The results were normalized to the levels of β-actin. **c** Western blotting of ZEB1 protein expression in MDA-MB-231 and SUM-159 cells after treatment with MG132 in the presence or absence of palbociclib. **d** Coimmunoprecipitation analysis of ZEB1 protein ubiquitination in MDA-MB-231 and SUM-159 cells after treatment with palbociclib for 48 h. The cells were treated with MG132 for 12 h prior to harvest. **e** Western blotting of EMT markers in MDA-MB-231 and SUM-159 cells treated with palbociclib for 48 h. **f**, **g** Transwell migration (**f**) and wound-healing (**g**) assays in ZEB1-expressing MDA-MB-231 and SUM-159 cells treated with palbociclib. Scale bars, 100 μm. **P *<* 0.01, ***P *<* 0.001 vs. respective control by unpaired Student’s *t*-test. **h** 3D outgrowth invasion assay in ZEB1-expressing MDA-MB-231 cells treated with palbociclib. Scale bars, 20 μm. **P *<* 0.01 vs. respective control by unpaired Student’s *t*-test. **i**, **j** Representative images and the quantification of lung nodules of BALB/c nude mice that were injected with ZEB1-expressing MDA-MB-231 cells and treated with palbociclib. Scale bars, 100 and 20 μm. *P *<* 0.05, **P *<* 0.01, ***P *<* 0.001 vs. respective controls by unpaired Student’s *t*-test.

Furthermore, Western blotting assays showed that CDK4/6 inhibition in response to palbociclib significantly decreased ZEB1 protein levels in a dose-dependent manner (Fig. 1a); however, the quantitative PCR results revealed that palbociclib had no effect on ZEB1 mRNA expression (Supplementary Fig. S2a). Moreover, the combined treatment with palbociclib and CHX resulted in an increased degradation of ZEB1 protein in MDA-MB-231 and SUM-159 cells (Fig. 1b), and remarkably, this effect was abolished by the addition of MG132 (Fig. 1c) but not CQ (Supplementary Fig. S2b). The ubiquitination assay further revealed that treatment with palbociclib increased the polyubiquitination levels of ZEB1 protein in the presence of MG132 (Fig. 1d). Furthermore, Lee011, which is another highly selective CDK4/6 inhibitor, was used to verify that the specific inhibition of CDK4/6 kinase activity promoted ZEB1 protein degradation in a ubiquitin-proteasome-dependent manner in MDA-MB-231 and SUM-159 cells (Supplementary Fig. S2c–h).

In addition, the wild-type variants of CDK4/6 and their hyperactive mutant forms, CDK4-R24C^31^ and CDK6-R31C,^32^ were overexpressed in MDA-MB-231 and SUM-159 cells, which led to a significant upregulation of ZEB1 protein expression (Supplementary Fig. S3a). In contrast, CDK4/6 depletion by specific shRNAs had the opposite effect and decreased ZEB1 protein levels (Supplementary Fig. S3b). Mechanistically, the knockdown of CDK4/6 was confirmed to induce ZEB1 protein degradation via the ubiquitin-proteasome pathway, which is an effect that is similar to that seen with treatment with palbociclib or Lee011 (Supplementary Fig. S3c–h).

### CDK4/6 inhibition prevents cancer metastasis through the regulation of ZEB1

ZEB1 plays pivotal roles in the induction of EMT and cancer metastasis, which led us to examine whether CDK4/6 inhibition could regulate cancer metastasis via ZEB1. As shown in Fig. 1e, Western blotting showed that CDK4/6 kinase inhibition by palbociclib increased the expression of E-cadherin (an epithelial marker) but decreased the expression of vimentin and N-cadherin (mesenchymal markers). Furthermore, transwell (Fig. 1f), wound-healing (Fig. 1g) and 3D outgrowth invasion (Fig. 1h) assays demonstrated that treatment with palbociclib inhibited cell migration and invasion in MDA-MB-231 and SUM-159 cells; however, this effect was significantly weakened by the rescue of ZEB1 expression. In addition, depletion of CDK4/6 activity by treatment with Lee011 and shRNA knockdown was used to verify that CDK4/6 inhibition reversed the EMT phenotype and prevented tumor cell metastasis through the regulation of ZEB1 in vitro (Supplementary Figs. S4, S5).

We further investigated the effects of CDK4/6 inhibition on cancer cell metastasis in a xenograft metastasis model. To do so, ZEB1-expressing or control cells were injected into the mammary fat pad of female BALB/c nude mice followed by treatment with palbociclib. We found that palbociclib treatment markedly inhibited lung metastases in control mice. However, this effect was attenuated in mice carrying ZEB1-expressing tumors (Fig. 1i, j), showing that CDK4/6 inhibition resulted in reduced cancer metastasis through the regulation of ZEB1 in vivo.

### USP51 deubiquitinates and stabilizes ZEB1

**Fig. 2:**
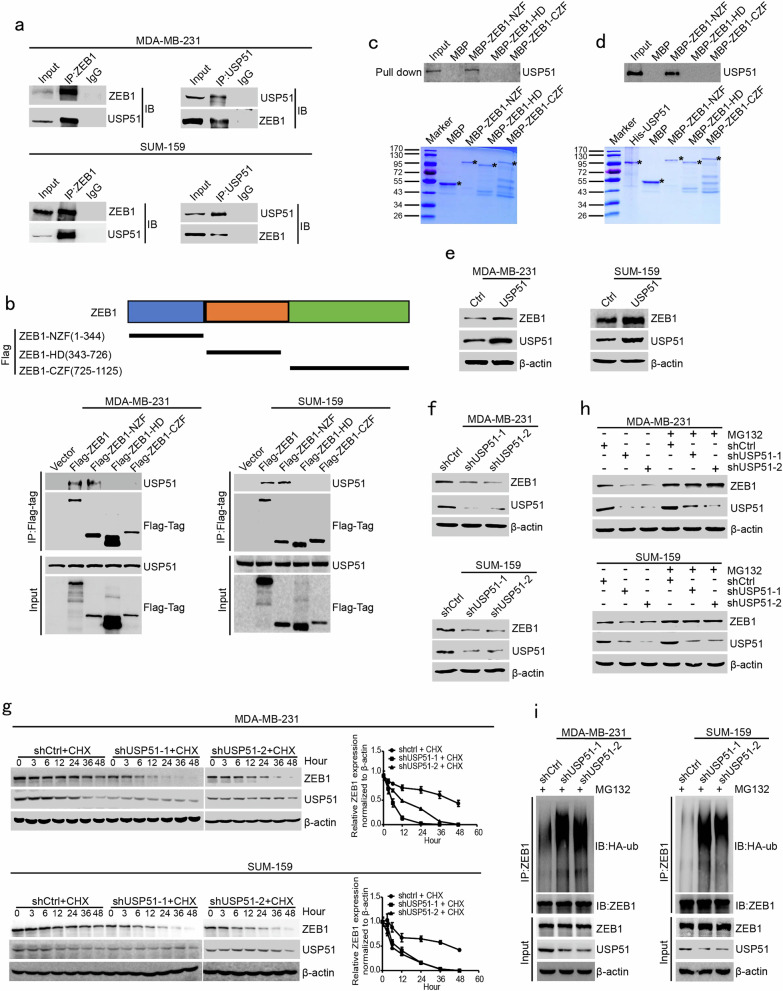
USP51 deubiquitinates and stabilizes ZEB1. **a** Coimmunoprecipitation analysis of the interaction between USP51 and ZEB1 in MDA-MB-231 and SUM-159 cells. **b** Coimmunoprecipitation analysis of the interaction between USP51 and wild-type ZEB1 (Flag-ZEB1) or deletion mutants (Flag-ZEB1-NZF, Flag-ZEB1-HD, and Flag-ZEB1-CZF) of ZEB1 in MDA-MB-231 and SUM-159 cells. **c** MBP pulldown analysis of the interaction between USP51 and the deletion mutants (MBP-ZEB1-NZF, MBP-ZEB1-HD, and MBP-ZEB1-CZF) in MDA-MB-231 cells. **d** In vitro binding analysis of the interaction between His-USP51 and MBP-ZEB1-NZF purified recombinant proteins. **e** Western blotting of USP51 and ZEB1 protein expression in USP51-expressing MDA-MB-231 and SUM-159 cells. **f** Western blotting of USP51 and ZEB1 protein expression in USP51-treated MDA-MB-231 and SUM-159 cells. **g** CHX pulse-chase analysis of ZEB1 protein stability in USP51-disrupted MDA-MB-231 and SUM-159 cells at the indicated time points. The results were normalized to the levels of β-actin. **h** Western blotting of ZEB1 protein expression in USP51-disrupted MDA-MB-231 and SUM-159 cells treated with MG132. **i** Coimmunoprecipitation analysis of ZEB1 protein ubiquitination in USP51-disrupted MDA-MB-231 and SUM-159 cells. The cells were treated with MG132 for 12 h prior to harvest.

It was previously reported that USP51, a deubiquitinase, could promote the deubiquitination and stabilization of ZEB1.^33^ Notably, we searched the PhosphoSitePlus® database and identified Ser26 as a major phosphorylation site on USP51 (Supplementary Fig. S6a), which also matches the CDK consensus motif S/T-P. To further validate the relationship between USP51 and ZEB1, we performed a coimmunoprecipitation (Co-IP) assay to demonstrate that USP51 physically interacted with full-length ZEB1 in MDA-MB-231 and SUM-159 cells (Fig. 2a). Three deletion constructs of ZEB1 (Flag-ZEB1-NZF, Flag-ZEB1-HD and Flag-ZEB1-CZF) were further generated (Fig. 2b). The Co-IP assays demonstrated that the deletion variant ZEB1-NZF, but not ZEB1-HD and ZEB1-CZF, was able to interact with USP51 (Fig. 2b). In addition, we constructed MBP fusion proteins for the deletion forms of ZEB1 to confirm the binding between ZEB1-NZF and USP51 in MDA-MB-231 cells (Fig. 2c). Importantly, an in vitro binding assay was performed to prove that the proteins purified from MBP-ZEB1-NZF and His-USP51 could physically interact under cell-free conditions, which demonstrates the direct binding of ZEB1-NZF and USP51 (Fig. 2d).

Next, we examined the role of USP51 in the regulation of ZEB1 expression. We showed that overexpression of USP51 elevated the expression of ZEB1 in MDA-MB-231 and SUM-159 cells (Fig. 2e). In contrast, the knockdown of USP51 by specific shRNAs decreased ZEB1 protein expression (Fig. 2f). Remarkably, USP51 depletion promoted ZEB1 protein degradation in response to treatment with CHX (Fig. 2g). However, this effect was rescued by the addition of MG132 (Fig. 2h). Taken together with the results of the ubiquitination assay showing that USP51 knockdown led to increased polyubiquitination of the ZEB1 protein upon MG132 treatment (Fig. 2i), the above observations collectively suggested that USP51 is a bona fide DUB that targets the ZEB1 protein for deubiquitination and stabilization.

### USP51 regulates cancer metastasis through ZEB1

**Fig. 3:**
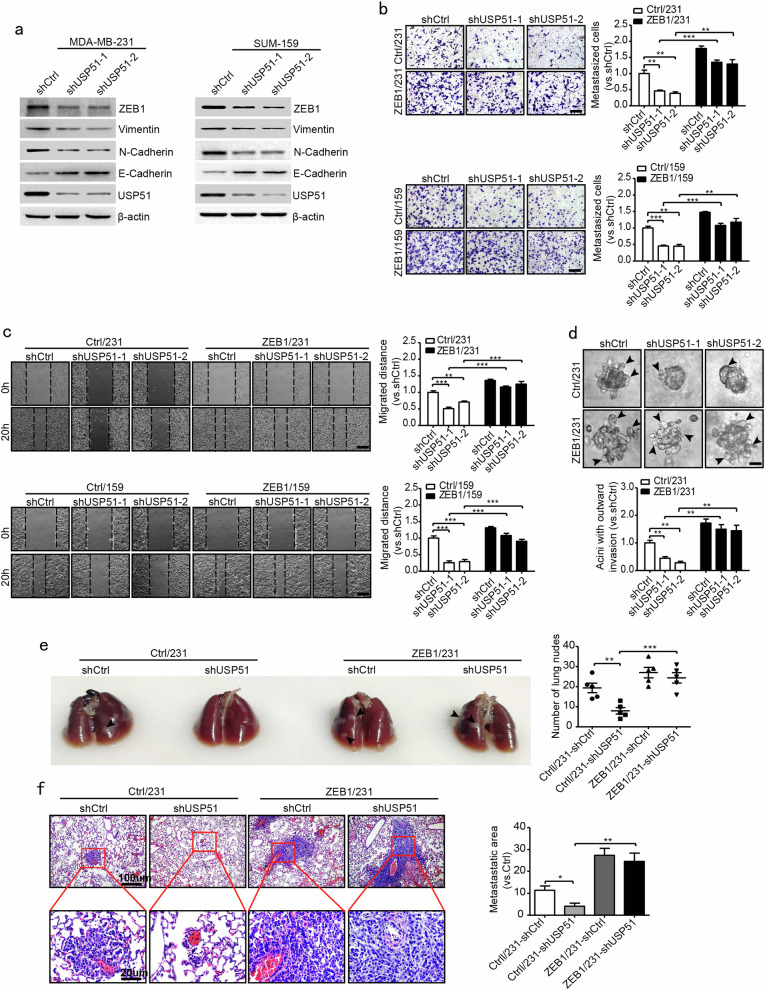
USP51 regulates breast cancer metastasis through ZEB1. **a** Western blotting of EMT markers in USP51-disrupted MDA-MB-231 and SUM-159 cells. **b**, **c** Transwell migration (**b**) and wound-healing (**c**) assays in USP51-disrupted MDA-MB-231 and SUM-159 cells in the presence or absence of rescued ZEB1 expression. Scale bars, 100 μm. **P *<* 0.01, ***P *<* 0.001 vs. respective control by unpaired Student’s *t*-test. **d** 3D outgrowth invasion assay in USP51-interfered MDA-MB-231 cells in the presence or absence of rescued ZEB1 expression. Scale bars, 20 μm. **P *<* 0.01 vs. respective control by unpaired Student’s *t*-test. **e**, **f** Representative images and quantification of lung nodules of BALB/c nude mice that were injected with USP51-interfered MDA-MB-231 cells in the presence or absence of rescued ZEB1 expression. Scale bars, 100 and 20 μm. *P *<* 0.05, **P *<* 0.01, ***P *<* 0.001 vs. respective control by unpaired Student’s *t*-test.

We next examined whether USP51 depletion could inhibit cancer cell metastasis through the regulation of ZEB1. Western blotting showed that the downregulation of USP51 resulted in increased E-cadherin expression but decreased vimentin and N-cadherin expression in MDA-MB-231 and SUM-159 cells (Fig. 3a). Moreover, the transwell (Fig. 3b), wound-healing (Fig. 3c) and 3D outgrowth invasion (Fig. 3d) assays demonstrated that USP51 interference significantly inhibited cell migration and invasion, which was abolished by ectopic ZEB1 expression.

We then investigated the effects of USP51 on cancer cell metastasis in a xenograft metastasis model. To do so, USP51-disrupted or control cells were injected into the mammary fat pad of female BALB/c nude mice in the presence or absence of ectopic ZEB1. We found that the knockdown of USP51 resulted in significantly decreased lung metastasis; however, this effect was attenuated in mice carrying ZEB1-expressing tumors (Fig. 3e, f), confirming that the knockdown of USP51 reduces cancer metastasis through the regulation of ZEB1 in vivo.

### CDK4/6 phosphorylates USP51 at Ser26

**Fig. 4:**
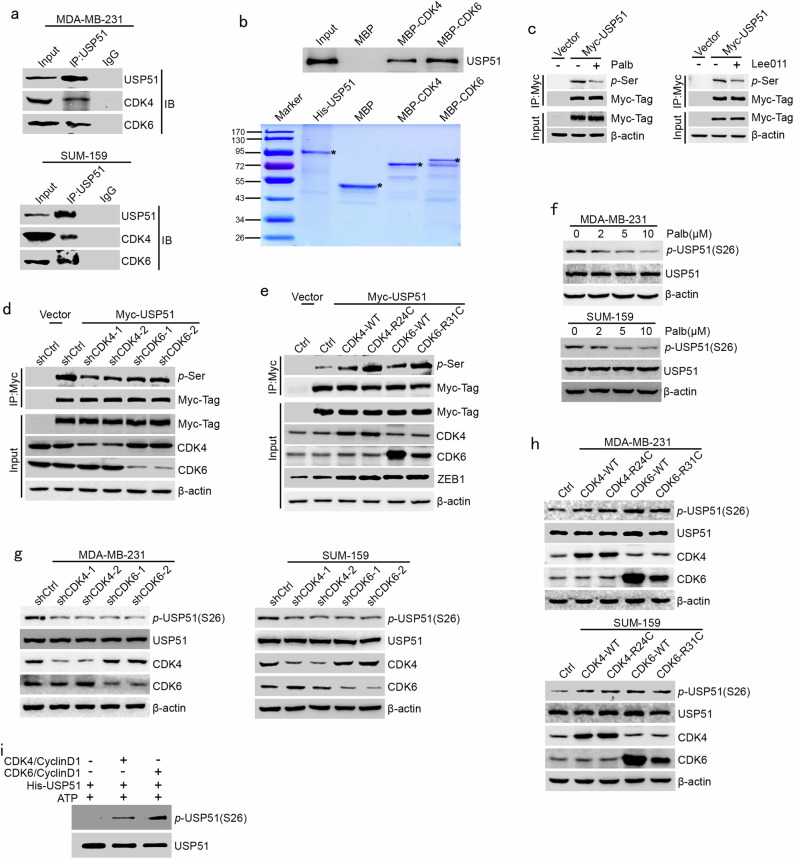
CDK4/6 phosphorylates USP51 at Ser26. **a** Coimmunoprecipitation analysis of the interaction between CDK4/6 and USP51 in MDA-MB-231 and SUM-159 cells. **b** In vitro binding analysis of the interaction between MBP-CDK4/6 and His-USP51 purified recombinant proteins. **c** Coimmunoprecipitation analysis of USP51 serine phosphorylation in 293T cells that were treated with palbociclib or Lee011 in the presence or absence of overexpressed Myc-USP51. **d** Coimmunoprecipitation analysis of USP51 serine phosphorylation in CDK4/6-disrupted 293T cells in the presence or absence of overexpressed Myc-USP51. **e** Coimmunoprecipitation analysis of USP51 serine phosphorylation in 293T cells overexpressing wild-type or hyperactive mutant forms (CDK4-R24C and CDK4-R31C) of CDK4/6 in the presence or absence of overexpressed Myc-USP51. **f** Western blotting of S26-USP51 phosphorylation in MDA-MB-231 and SUM-159 cells after treatment with the indicated concentrations of palbociclib for 48 h. **g** Western blotting of S26-USP51 phosphorylation in CDK4/6-disrupted MDA-MB-231 and SUM-159 cells. **h** Western blotting of S26-USP51 phosphorylation in MDA-MB-231 and SUM-159 cells overexpressing wild-type or hyperactive mutant forms (CDK4-R24C and CDK4-R31C) of CDK4/6. **i** In vitro analysis of S26-USP51 phosphorylation using the recombinant proteins His-USP51, CDK4/cyclin D1, and CDK6/cyclin D1 in the presence of ATP.

To investigate the link between CDK4/6 and USP51 in the regulation of ZEB1 protein stability, we performed an endogenous Co-IP assay that demonstrated the physical interactions between CDK4/6 and USP51 (Fig. 4a). Importantly, the in vitro binding assay confirmed that the proteins purified from His-USP51 and MBP-CDK4/6 were bound to each other under cell-free conditions, which suggests a direct interaction between USP51 and CDK4/6 (Fig. 4b).

To further examine whether CDK4/6 could phosphorylate the serine residues of USP51, we transfected 293T cells with Myc-tagged USP51 followed by treatment with palbociclib or Lee011 (Fig. 4c). The Co-IP assay revealed that the serine phosphorylation of USP51 was significantly decreased in the presence of CDK4/6 inhibitors. Similarly, the knockdown of CDK4/6 by shRNAs resulted in a similar effect on the reduction of the serine phosphorylation of USP51 in 293T cells (Fig. 4d); however, overexpression of the wild-type and hyperactive mutant forms of CDK4/6 showed the opposite effect (Fig. 4e).

To validate the hypothesis that Ser26-USP51 is a bona fide phosphorylation site for CDK4/6, we generated a phospho-Ser26-specific antibody for USP51. The Myc-tagged wild-type and Ser26-mutant USP51 were transfected into 293T cells. The Co-IP assay revealed that the *p*-USP51 (S26) antibody was able to specifically recognize the phosphorylation of Ser26-USP51 (Supplementary Fig. S6b). We next examined the CDK4/6-mediated phosphorylation of USP51 in MDA-MB-231 and SUM-159 cells. The results showed that the specific phosphorylation of Ser26-USP51 was largely reduced in the presence of palbociclib in a dose-dependent manner (Fig. 4f). Similar results were also obtained in response to the depletion of CDK4/6 (Fig. 4g). In contrast, overexpression of either the wild-type or hyperactive mutant forms of CDK4/6 exhibited the opposite effect and promoted Ser26-USP51 phosphorylation (Fig. 4h). Furthermore, Lee011 was used to verify that the specific inhibition of CDK4/6 kinase activity led to a strongly reduced phosphorylation of USP51 in MDA-MB-231 and SUM-159 cells (Supplementary Fig. S6c). Taken together with the results of the in vitro kinase assay, which showed that CDK4/6 directly phosphorylated USP51 protein at Ser26 under cell-free conditions (Fig. 4i), these observations provide evidence that USP51 is a direct substrate for CDK4/6 and that it might mediate the function of CDK4/6 to deubiquitylate and stabilize ZEB1.

### CDK4/6-induced phosphorylation of USP51 regulates ZEB1 protein degradation and cancer metastasis

**Fig. 5:**
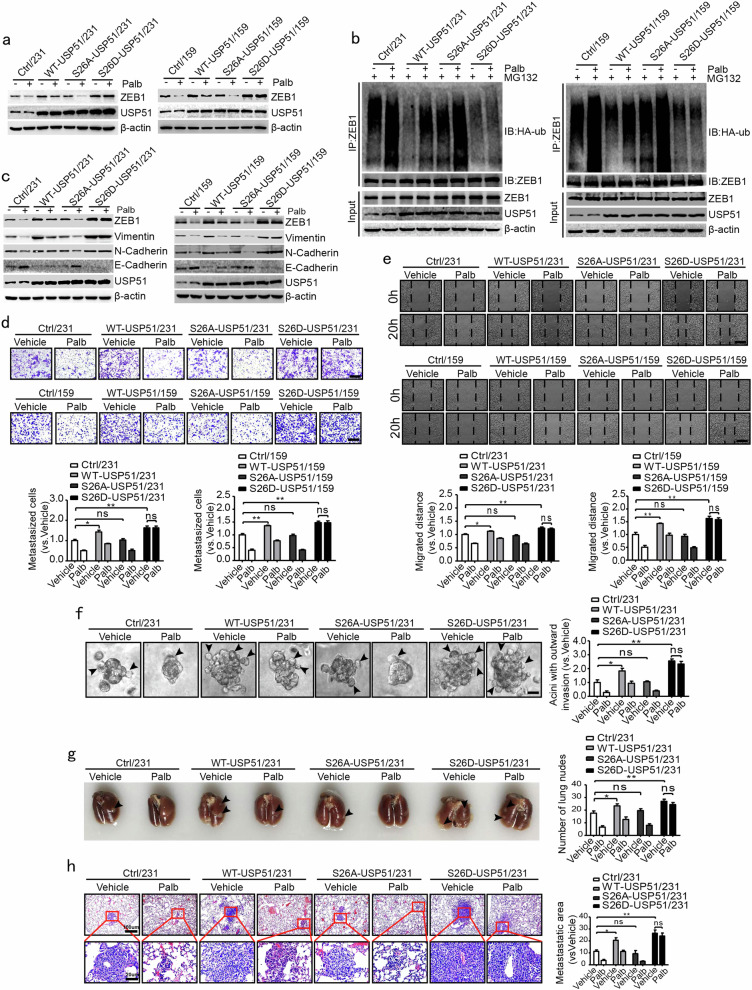
CDK4/6-mediated USP51 phosphorylation regulates ZEB1 protein degradation and cancer metastasis. **a** Western blotting of ZEB1 protein expression in MDA-MB-231 and SUM-159 cells overexpressing wild-type USP51 or two mutants (S26A-USP51 and S26D-USP51) of USP51 after treatment with palbociclib. **b** Coimmunoprecipitation analysis of ZEB1 protein ubiquitination in MDA-MB-231 and SUM-159 cells overexpressing wild-type USP51 or two mutants (S26A-USP51 and S26D-USP51) of USP51 after treatment with palbociclib. The cells were treated with MG132 for 12 h prior to harvest. **c** Western blotting of EMT markers in MDA-MB-231 and SUM-159 cells overexpressing wild-type USP51 or two mutants (S26A-USP51 and S26D-USP51) of USP51 after treatment with palbociclib. **d**, **e** Transwell migration (**d**) and wound-healing (**e**) assays in MDA-MB-231 and SUM-159 cells overexpressing the wild type or two mutants (S26A-USP51 and S26D-USP51) of USP51 by treatment with palbociclib. Scale bars, 100 μm. *P *<* 0.05, **P *<* 0.01 vs. respective control by unpaired Student’s *t*-test. **f** 3D outgrowth invasion assay in MDA-MB-231 cells overexpressing the wild type or two mutants (S26A-USP51 and S26D-USP51) of USP51 after treatment with palbociclib. Scale bars, 20 μm. *P *<* 0.05, **P *<* 0.01 vs. respective control by unpaired Student’s *t*-test. **g**, **h** Representative images and the quantification of lung nodules of BALB/c nude mice that were injected with MDA-MB-231 cells overexpressing wild-type USP51 or two mutants (S26A-USP51 and S26D-USP51) of USP51 after treatment with palbociclib. Scale bars, 100 and 20 μm. *P *<* 0.05, **P *<* 0.01 vs. respective control by unpaired Student’s *t*-test.

Next, we overexpressed wild-type USP51 and two mutants (S26A: dominantly negative; S26D: constitutively active) of USP51 in MDA-MB-231 and SUM-159 cells, followed by treatment with palbociclib. In contrast to wild-type USP51, ectopic expression of the dominant-negative mutant (S26A-USP51) did not upregulate ZEB1 protein levels regardless of palbociclib treatment (Fig. 5a), whereas the constitutively active mutant (S26D-USP51) produced the opposite effect and constantly promoted the expression of ZEB1 protein. The ubiquitination assay further revealed that overexpression of wild-type and S26D-USP51 resulted in a large reduction in the polyubiquitination levels of ZEB1 in the absence of palbociclib, but this effect was not shown for S26A-USP51 (Fig. 5b). Notably, CDK4/6 inhibition by palbociclib resulted in increased ubiquitination of the ZEB1 protein in wild-type- and S26A-USP51-expressing cells; however, this effect was remarkably attenuated in S26D-USP51-expressing cells (Fig. 5b). These results collectively suggested that the Ser26 phosphorylation site on USP51 is critical for the CDK4/6-regulated protein stability of ZEB1.

Western blotting assays further showed that unlike overexpression of wild-type USP51, S26A-USP51 overexpression did not alter the expression of EMT markers regardless of palbociclib treatment in MDA-MB-231 and SUM-159 cells (Fig. 5c). In contrast, ectopic S26D-USP51 resulted in the constant upregulation of vimentin and N-cadherin but downregulated E-cadherin (Fig. 5c), which shows that the CDK4/6-induced phosphorylation of Ser26-USP51 plays a pivotal role in the regulation of EMT through the deubiquitylation and stabilization of ZEB1. In addition, we performed transwell (Fig. 5d), wound-healing (Fig. 5e) and 3D outgrowth invasion (Fig. 5f) assays to demonstrate that the phosphorylation of Ser26-USP51 is able to mediate the cell migration and invasion induced by CDK4/6 in breast cancer cells in vitro.

We further investigated the function of CDK4/6-mediated phosphorylation of Ser26-USP51 in a xenograft metastasis model. The results showed a significant reduction in lung metastasis in mice that carried MDA-MB-231 tumors reconstituted with vectors for WT-USP51 or S26A-USP51 in response to palbociclib treatment, and this effect was abolished in tumors reconstituted with S26D-USP51 (Fig. 5g, h). Altogether, these results demonstrate that the phosphorylation of Ser26-USP51 is critical for CDK4/6-mediated breast cancer metastasis in vivo.

### The expression of *p*-USP51 and ZEB1 is positively correlated with CDK4/6 activity in human breast cancer patients

**Fig. 6:**
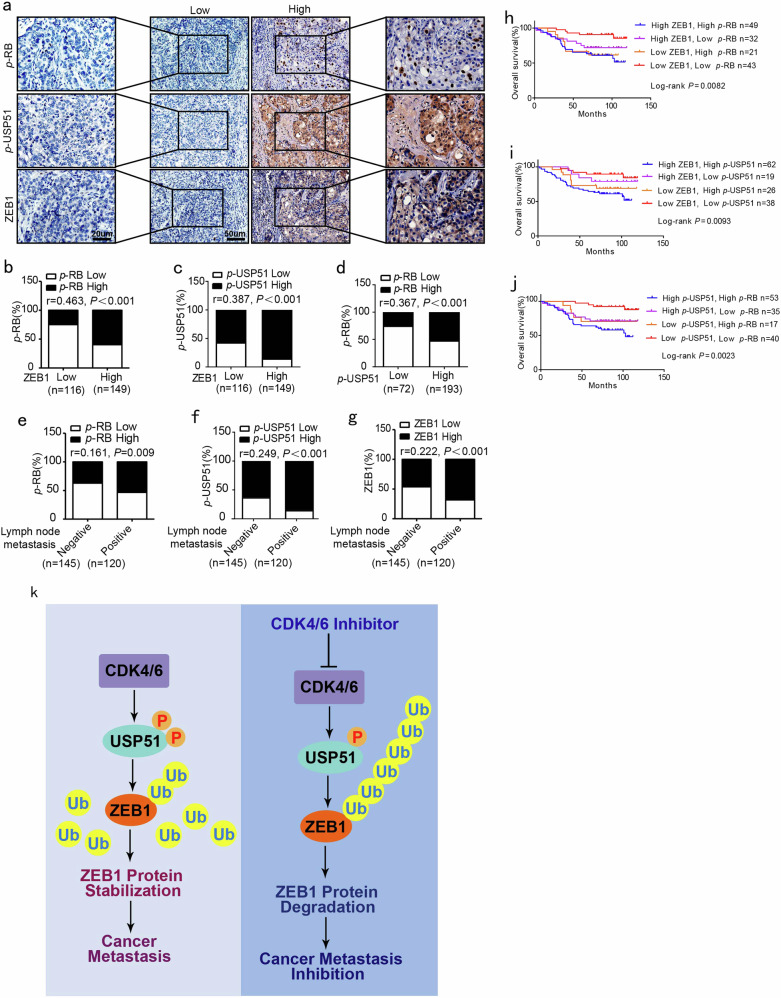
The expression of *p*-USP51 and ZEB1 is positively correlated with CDK4/6 activity in human breast cancer. **a** Representative images of the immunohistochemical staining of *p*-RB, *p*-USP51, and ZEB1 in serial sections of the same tumor from two patients. Scale bars, 20 μm and 50 μm. **b** A positive correlation between the expression of *p*-RB and ZEB1 in 265 human breast cancer samples. *r* = 0.463, *P* *<* 0.001 by Spearman’s rank correction test. **c** A positive correlation between the expression of *p*-USP51 and ZEB1 in 265 human breast cancer samples. *r* = 0.387, *P* *<* 0.001 by Spearman’s rank correction test. **d** A positive correlation between the expression of *p*-RB and *p*-USP51 in 265 human breast cancer samples. *r* = 0.367, *P* *<* 0.001 by Spearman’s rank correction test. **e** Increased expression of *p*-RB in metastatic breast cancer. *r* = 0.161, *P* *=* 0.009 by chi-square test. **f** Increased expression of *p*-USP51 in metastatic breast cancer. *r* = 0.249, *P* *<* 0.001 by chi-square test. **g** Increased expression of ZEB1 in metastatic breast cancer. *r* = 0.222, *P* *<* 0.001 by chi-square test. **h** Kaplan–Meier curves showing that patients with concomitantly high expression of *p*-RB and ZEB1 in their breast tumors have shorter overall survival than patients with concomitantly low expression. *P* *=* 0.0082 by log-rank test. **i** Kaplan–Meier curves showing that patients with concomitantly high expression of *p*-USP51 and ZEB1 in their breast tumors have shorter overall survival than patients with concomitantly low expression. *P* = 0.0093 by log-rank test. **j** Kaplan–Meier curves showing that patients with concomitantly high expression of *p*-RB and *p*-USP51 in their breast tumors have shorter overall survival than patients with concomitantly low expression. *P* = 0.0023 by log-rank test. **k** A working model to illustrate that CDK4/6-USP51-dependent deubiquitination and ZEB1 protein stability regulate breast cancer EMT and metastasis.

To further examine the pathological correlations between *p*-USP51, ZEB1, and CDK4/6 activity, we performed immunohistochemical staining for *p*-RB, *p*-USP51, and ZEB1 in 265 cases of human primary breast carcinoma. As shown in Fig. 6a, the subjects were divided into two groups based on their ZEB1 expression scores. The results demonstrated a strong positive correlation between the expression of *p*-RB, *p*-USP51, and ZEB1 (Fig. 6b–d). We also observed increased expression of *p*-RB, *p*-USP51, and ZEB1 in tumors with lymph node metastasis (Fig. 6e–g). Notably, the survival analysis indicated that cancer patients with concomitantly high expression of *p*-RB, *p*-USP51, and ZEB1 in their tumors had shorter overall survival than those with low *p*-RB, *p*-USP51, and ZEB1 expression (Fig. 6h–j). In addition, elevated expression of ZEB1 and total USP51 protein was demonstrated to be correlated with poor overall survival rates in the same cohort (Supplementary Fig. S7), which is consistent with the notion that ectopic USP51 contributes to the malignant progression of breast cancer.^33^ Altogether, these observations reveal that aberrant functionality of the CDK4/6-USP51-ZEB1 axis might contribute to metastasis and could be used to predict poor clinical outcomes in breast cancer patients.

## Discussion

A growing body of evidence suggests that ZEB1 promotes tumor initiation and malignant progression in breast cancer patients.^5,6^ Therefore, identifying the signaling pathways that regulate ZEB1 stabilization may lead to the development of improved antineoplastic therapies. Based on our findings, we proposed that the inhibition of CDK4/6 activity blocks breast cancer EMT and metastasis by decreasing the protein stability of ZEB1. Mechanistically, we identified the deubiquitinase USP51 as a bona fide target of CDK4/6, and the CDK4/6-mediated phosphorylation and activation of USP51 was found to be essential for deubiquitinating and stabilizing ZEB1. Thus, our study established the CDK4/6-USP51-ZEB1 axis as an important regulatory mechanism of breast cancer metastasis and provided a rationale for future therapeutic interventions in the treatment of advanced breast cancer (Fig. 6k).

It is well established that CDK4/6 inhibitors target the cyclin D/CDK/retinoblastoma signaling pathway, inducing cell cycle arrest, decreasing cell viability and causing tumor shrinkage.^34^ As the cyclin D/CDK complex is activated downstream of estrogen signaling, the combination of CDK4/6 inhibitors with standard endocrine therapies is a rational approach to elicit synergistic antitumor activity in estrogen receptor (ER)-positive breast cancer. Recently, selective CDK4/6 inhibitors have shown impressive results when combined with endocrine therapies (e.g., letrozole and fulvestrant) in advanced breast cancer, leading to the FDA approval of three compounds: palbociclib, ribociclib and, most recently, abemaciclib.^35,36^ Of note, the antimetastatic activity that is triggered by CDK4/6 inhibition can be regulated independently from primary tumor development.^37^ However, the underlying mechanisms are not clearly understood. In the present study, we reported that the inhibition of CDK4/6 significantly reduced breast cancer EMT and metastasis through the induction of ZEB1 protein degradation. Furthermore, our data indicate the potential use of CDK4/6 inhibitors in the treatment of triple-negative breast cancer (TNBC). Consistent with previous studies,^37^ we showed that CDK4/6 inhibition with palbociclib and Lee011 did not affect the growth of primary tumors derived from human TNBC cells (data not shown). Instead, treatment with palbociclib and Lee011 induced the inactivation of USP51, the destabilization of ZEB1 protein and a decrease in cell migration, thereby reducing the degree of TNBC metastasis both in vitro and in vivo. Given that TNBC with elevated ZEB1 expression is particularly aggressive and more likely to metastasize,^38^ our study indicated a new potential paradigm in the treatment of TNBC metastasis through the use of CDK4/6 inhibitors. Beyond breast cancer, a promising role for CDK4/6 inhibitors has also been observed in other malignancies, including NSCLC, glioblastoma (GBM), melanoma, and colorectal and ovarian cancers,^39,40^ which suggests their potential efficacy across multiple tumor types.

During cancer progression, the ubiquitylation and deubiquitylation systems play critical roles in aberrant signaling amplification, promoting survival and coping with DNA repair.^20,41^ Specifically, several recent studies have revealed that USP51 regulates the DNA damage response as well as tumor growth. For example, ectopic USP51 correlates with a shorter overall survival rate and poorer outcomes in breast and colon cancer patients.^33^ Here, our study expanded on the multifaceted role of USP51 in cancer progression by demonstrating its function as a key metastasis activator through the promotion of ZEB1-mediated EMT, migration and metastasis. Mechanistically, USP51 phosphorylation of Ser26 by CDK4/6 is critical for the activation of its enzymatic function. Thus, a dominant-negative mutant (S26A) impairs the catalytic activity of USP51 and fails to alter ZEB1 protein stability regardless of CDK4/6 activity. We found that Ser26 is located in an unstructured region of the protein, which is outside of the ubiquitin hydrolase domain.^42^ These posttranslational modifications may activate or modulate the regulatory subunits of USP51, which in turn controls its deubiquitinase activity and possibly substrate specificity.^43^ In addition, USP51 phosphorylation may trigger structural changes and affect ubiquitin recognition. Further detailed structural studies on USP51 should be carried out to validate the role of phosphorylation in its catalytic activity.

In line with our results, the expression of ZEB1 is suggested to be tightly regulated at the transcriptional and posttranscriptional levels.^5,6^ Various cellular cues, such as TGF-β, Wnt and miR-200 family members, can transcriptionally modulate ZEB1 mRNA expression, thus inducing EMT and tumorigenicity in mammalian cells.^16^ On the other hand, posttranslational modifications have also been identified to regulate ZEB1 protein levels. For example, the ubiquitin ligase Siah1/2 and the Skp1-Pam-Fbxo45 complex have been shown to promote ZEB1 ubiquitination and degradation.^18,44,45^ Moreover, Zhang et al. found that ATM is rapidly activated upon radiation exposure, which phosphorylates and stabilizes ZEB1 protein in radioresistant breast cancer cells.^19^ However, the molecular mechanisms by which the ZEB1 protein is stabilized remain unclear. In the present study, we provided evidence that USP51 is a bona fide DUB that targets the ZEB1 protein for deubiquitination and stabilization, which is crucial for the induction of EMT and metastasis in breast cancer. In a subset of human breast cancer cell lines and patient samples, the status of USP51 is correlated with ZEB1 expression. Interestingly, the correlation between USP51 and ZEB1 is predominantly present in metastatic breast cancer. Given our observation that USP51 directly binds and deubiquitinates ZEB1, it follows that disrupting the interaction between USP51 and ZEB1 would severely diminish ZEB1 activity and thus the aggressiveness of breast cancer in vitro and in vivo.

In summary, we demonstrated an alternative mechanism for the CDK4/6-USP51 axis in regulating breast cancer metastasis that supplements the deubiquitination and stabilization of ZEB1. Given that the EMT-inducing transcription factor ZEB1 plays an essential role in cancer cell plasticity and tumor recurrence, future studies should focus on the role CDK4/6-USP51 signaling plays in breast cancer initiation, aggressiveness and therapy resistance through ZEB1. Importantly, because DUBs are amenable to pharmacological inhibition by small molecule inhibitors,^46^ targeting USP51 to reduce ZEB1 stability in conjunction with CDK4/6 inhibitors represents a new therapeutic strategy that could deplete ZEB1 protein and overcome metastasis and therapy resistance in breast cancer.

## Materials and methods

### Cell culture

MDA-MB-231 and 293T cell lines were obtained from the American Type Culture Collection (Manassas, VA, USA), and the SUM-159 cell line was obtained from Asterand Bioscience (Detroit, MI, USA). Each cell line was recently authenticated and was not contaminated by mycoplasma. MDA-MB-231 cells were cultured with RPMI-1640 supplemented with 10% fetal bovine serum (FBS) at 37 °C in 5% CO_2_. SUM-159 and 293T cells were cultured with high-glucose Dulbecco’s modified Eagle’s medium (DMEM) supplemented with 10% fetal bovine serum (FBS) at 37 °C in 5% CO_2_.

### Lentiviral knockdown and expression systems

Specific shRNAs for CDK4/6 and USP51 were annealed and subcloned into the pLV-H1-EF1α-puro vector (Biosettia, San Diego, CA, USA). CDK4/6, ZEB1, USP51 or their respective mutants were subcloned into the pLV-EF1-MCS-IRES-Bsd vector (Biosettia, San Diego, CA, USA). 293T cells were then cotransfected with the constructs and packaging mix using the Lipofectamine 2000 reagent (Invitrogen, Carlsbad, CA, USA) to generate lentiviral particles. The primer sequences are listed in Table S2.

### Western blotting assay

Preparation of the total cell extracts and western blotting with appropriate antibodies were performed as previously described.^47^ The antibodies used are listed in Table S3.

### RNA extraction and quantitative RT-PCR

Cells were treated with biochanin A, palbociclib or Lee011 for 48 h. Total RNA from each sample was isolated using TRIzol reagent (Invitrogen, Carlsbad, CA, USA) and used for first-strand cDNA synthesis using M-MLV Reverse Transcriptase (Takara, Kusatsu, Japan). ZEB1 was specifically amplified using quantitative PCR with the TransStart Green Q-PCR SuperMix kit (TransGen, Beijing, China). GAPDH was used as a normalization control. The primer sequences are listed in Table S2.

### Cellular thermal shift assay

The cellular thermal shift assay was performed as previously described^48^ with some modifications. Briefly, cells were incubated in a high concentration of biochanin A or palbociclib for 3 h before being detached with trypsin and collected in PBS that contained a complete protease inhibitor cocktail. Cells were then divided into four aliquots and heated individually at 40, 43, 46, or 49 °C for 3 min. Subsequently, the cell suspensions were freeze-thawed three times using liquid nitrogen. The soluble proteins were separated from the precipitated fraction by centrifugation at 20,000 × *g* for 20 min. The proteins were then detected by a western blotting assay using CDK4 and CDK6 antibodies.

### Deubiquitination assay

Cells were transfected with HA-ubiquitin plasmids, followed by treatment with the proteasome inhibitor MG132 for 12 h. The cell lysates were prepared in RIPA buffer and incubated with an anti-ZEB1 antibody or IgG at 4 °C overnight. The immunoprecipitates were then used in Western blotting assays with an anti-HA-Tag antibody.

### Wound-healing assay

Cells were allowed to grow to full confluence in a 6-well plate, and a wound was then created using a 10 μL pipette tip. The complete medium was replaced with serum-free medium, followed by treatment with palbociclib. The images were photographed under a light microscope (Olympus, Tokyo, Japan), and the migration distance was quantified using ImageJ software.

### Transwell assay

Cells were placed in the upper chambers and allowed to migrate, followed by treatment with palbociclib. After 20 h, the nonmigrated cells were scraped with a cotton swab. The migrated cells were then fixed with 20% methanol and stained with 0.5% crystal violet. The stained cells were counted and photographed under a light microscope (Olympus, Tokyo, Japan).

### 3D outgrowth assay

The 3D outgrowth assay was performed as previously described.^49^ Briefly, wells were coated with 250 μL of cold Matrigel and incubated at 37 °C for 30 min. Cells were harvested and pelleted at 1600 rpm for 2 min before being resuspended in cold Matrigel (4 × 10^4^ cells/0.2 ml) and quickly added to a preset Matrigel layer and then being allowed to set via incubation at 37 °C for 30 min. Once the gel was set, 500 μL of RPMI-1640 supplemented with 10% FBS was carefully added to each well and incubated at 37 °C for 2 weeks. The cultures were photographed under a light microscope (Olympus, Tokyo, Japan).

### Tumor xenograft experiments

All of the experimental procedures involving animals were performed in accordance with a protocol that was approved by the Ethics Committee for Animal Use at the Medical College of Nankai University. Six-week-old female BALB/c nude mice were used. Cells were injected into the mammary fat pads of the mice. When the tumors reached 300 mm^3^ in size, the primary tumors were removed, and the mice were treated with palbociclib (100 mg/kg, once per 2 days, PO) for an additional 12 weeks. The mice were then sacrificed, and the number of metastatic lung nodules was analyzed.

### Coimmunoprecipitation and pulldown assays

Cells were harvested and lysed in RIPA buffer on ice for 30 min. For the pulldown assay, the cell lysates were incubated with MBP-tagged proteins and AmyloseResin beads overnight at 4 °C. For the coimmunoprecipitation assay, the cell lysates were precleared with protein G Dynabeads (Invitrogen, Carlsbad, CA, USA) and incubated with the primary antibody or IgG at 4 °C overnight prior to being incubated with protein G Dynabeads (Invitrogen, Carlsbad, CA, USA) for 4 h. The beads were washed with lysis buffer three times, and the immunoprecipitates were then used in western blotting analysis.

### In vitro phosphorylation assay

The in vitro phosphorylation assay reactions were carried out using recombinant CDK4/cyclin D1 and CDK6/cyclin D1 protein (0.1–1 µg, Abcam, Cambridge, MA, USA) along with purified His-USP51 (0.25 µg) as the substrate, cold ATP (0.2 mM) and 1× kinase buffer (Cell Signaling Technology, Danvers, MA, USA) in a total volume of 30 µL. The reaction was carried out at 30 °C for 30 min and then stopped by adding 10 μL of 4× SDS sample buffer. The proteins were then used in Western blotting assays with a *p*-USP51(S26) antibody.

### In vitro binding assays

Purified His-USP51 was incubated with purified MBP-ZEB1 or MBP-CDK4/6 at 4 °C overnight. AmyloseResin beads were washed with wash buffer. The bound proteins were then eluted in boiling 2× SDS sample buffer and used in western blotting analysis.

### Tissue microarray and immunohistochemistry (IHC) scoring

Twenty fresh breast carcinoma tissues were obtained from the First Affiliated Hospital of Chongqing Medical University (Chongqing, China) and the study was approved by the Ethics Committee of the First Affiliated Hospital of Chongqing Medical University, tissue microarrays containing 100 breast carcinoma tissues were obtained from Alenabio Biotechnology Co., Ltd., Xi’an, China (catalog number: BC081120c), and tissue microarrays containing 145 breast carcinoma tissues with overall survival rate were obtained from Shanghai Outdo Biotech Co., Ltd., China. Written informed consent for using clinical information and tissue samples was obtained from all patients. All of the patients had histologically confirmed breast carcinoma (Tables S4–S7). The samples were stained with ZEB1 (ab87280, Abcam, Cambridge, MA, USA), USP51 (SAB1305451, Sigma-Aldrich, St. Louis, MO, USA), *p*-USP51 (custom order, Abclonal, Wuhan, China) and *p*-RB (8516S, Cell Signaling Technology, Danvers, MA, USA) antibodies using the Envision Kit (Dako, Beijing, China) following the manufacturer’s protocol. The immunostaining was independently evaluated by two pathologists. The IHC score was calculated by combining the quantity score (the percentage of positive-stained areas) with the staining intensity score. The quantity score ranges from 0 to 4: 0, no immunostaining; 1, 1–14% of the areas are positive; 2, 15–49% of the areas are positive; 3, 50–74% of the areas are positive; and 4, ≥75% of the areas are positive. The staining intensity was scored as follows: 0 (no color), 1 (light yellow), 2 (light brown), 3 (brown), and 4 (dark brown). The score for each tissue was calculated by summing the intensity and quantity scores (the range of this calculation was therefore 0–8). Samples with an IHC score >4 were classified as high expression, and those with an IHC score ≤4 were classified as low expression.

### Statistical analysis

SPSS 17.0 software (SPSS, IBM, USA) was used for the statistical analysis. The data from all of the experiments are presented as the means ± SD and represent three independent experiments. A Spearman correlation test was used to analyze the correlation of gene expression in tissue samples. Student’s *t*-test was used for the unpaired observations. A *P*-value < 0.05 was considered significant.

## Supplementary information

Supplementary information
