# Correction to: CDK4/6 inhibition blocks cancer metastasis through a USP51-ZEB1-dependent deubiquitination mechanism

**DOI:** 10.1038/s41392-020-00212-9

**Published:** 2020-06-19

**Authors:** Zhen Zhang, Jianjun Li, Yang Ou, Guang Yang, Kaiyuan Deng, Qiong Wang, Zhaoyang Wang, Wenhao Wang, Quansheng Zhang, Hang Wang, Wei Sun, Peiqing Sun, Shuang Yang

**Affiliations:** 1 Tianjin Key Laboratory of Tumor Microenvironment and Neurovascular Regulation, Medical College of Nankai University, Tianjin, 300071 China; 2 College of Pharmacy, Nankai University, Tianjin, 300071 China; 3 Tianjin Key Laboratory of Organ Transplantation, Tianjin First Center Hospital, Tianjin, 300192 China; 4 Department of Cancer Biology, Wake Forest University School of Medicine, Winston-Salem, NC 27157 USA

**Keywords:** Breast cancer, Epigenetics

Correction to: *Signal Transduction and Targeted Therapy*10.1038/s41392-020-0118-x, published online 11 March 2020.

Recently, in the process of collating the raw data, the authors noticed two inadvertent mistakes in Fig. 1 that need to be corrected.^1^ The correct data are provided as follows. The key findings of the article are not affected by these corrections.

1. During the preparation of Fig. 1e, the image representing showing vimentin and E-cadherin expression in SUM-159 cells was made a mistake when pasted the picture, which is an inadvertent mistake. After checked the original data, we feel the figure should be as shown here.

**Figure 1:**
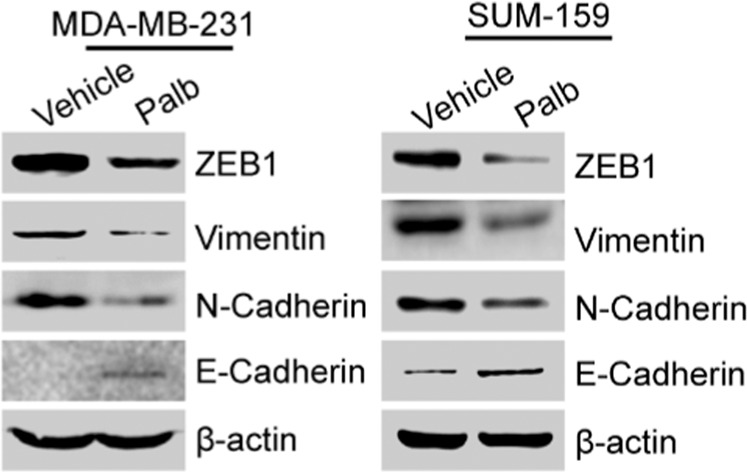
Fig. 1e
2. In the right panel of Fig. 1h, the pillar color of ZEB1/231 cells under Palb treatment was inadvertently made a mistake. After checked the original data, we feel the figure should be as shown here.

**Figure 2:**
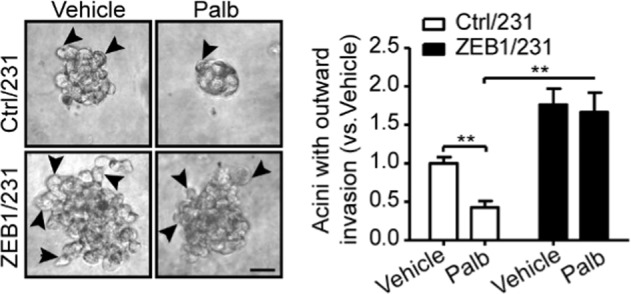
Fig. 1h
