# Correction: CDK4/6 inhibition blocks cancer metastasis through a USP51-ZEB1-dependent deubiquitination mechanism

**DOI:** 10.1038/s41392-024-01972-4

**Published:** 2024-10-04

**Authors:** Zhen Zhang, Jianjun Li, Yang Ou, Guang Yang, Kaiyuan Deng, Qiong Wang, Zhaoyang Wang, Wenhao Wang, Quansheng Zhang, Hang Wang, Wei Sun, Peiqing Sun, Shuang Yang

**Affiliations:** 1 Tianjin Key Laboratory of Tumor Microenvironment and Neurovascular Regulation, Medical College of Nankai University, Tianjin, 300071 China; 2 College of Pharmacy, Nankai University, Tianjin, 300071 China; 3 Tianjin Key Laboratory of Organ Transplantation, Tianjin First Center Hospital, Tianjin, 300192 China; 4 Department of Cancer Biology, Wake Forest University School of Medicine, Winston-Salem, NC 27157 USA

Correction to: *Signal Transduction and Targeted Therapy* (2020) **5**:25; 10.1038/s41392-020-0118-x, published online 11 March 2020

The authors noticed one inadvertent mistake in Supplementary Figure S5d that needs to be corrected. The image of shCDK6-1 (20h) in the ZEB1/231 group was repeatedly inserted as the image of shCDK4-2 (20h) by mistake during the preparation of the figures. The correct image of shCDK4-2 (20h) in the ZEB1/231 group is provided as follows. The accompanying quantification of Supplementary Figure S5d was not affected by this mistake, since the authors previously used the correct raw images to perform statistical analysis. The key findings of the article are not affected by these corrections.
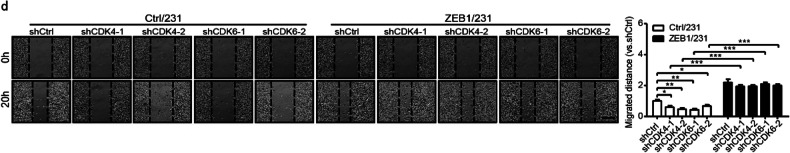


Supplementary Figure. S5d. Wound healing analysis in CDK4/6-interfered MDA-MB-231 cells in the presence or absence of rescued ZEB1 expression. *P < 0.05, **P < 0.01, ***P < 0.001 vs. respective control by unpaired Student’s t-test.

The authors noticed one inadvertent mistake that occurred during the preparation of Figure 1d (left panel) that needs to be corrected. The band of ZEB1 in the IP:ZEB1 group (left panel of Supplementary Figure S1g) was repeatedly inserted as the band of ZEB1 in the Input group (left panel of Figure 1d) by mistake during the preparation of the figures. The correct band of ZEB1 in the Input group (left panel of Figure 1d) is provided as follows. The key findings of the article are not affected by these corrections. The original article has been corrected.
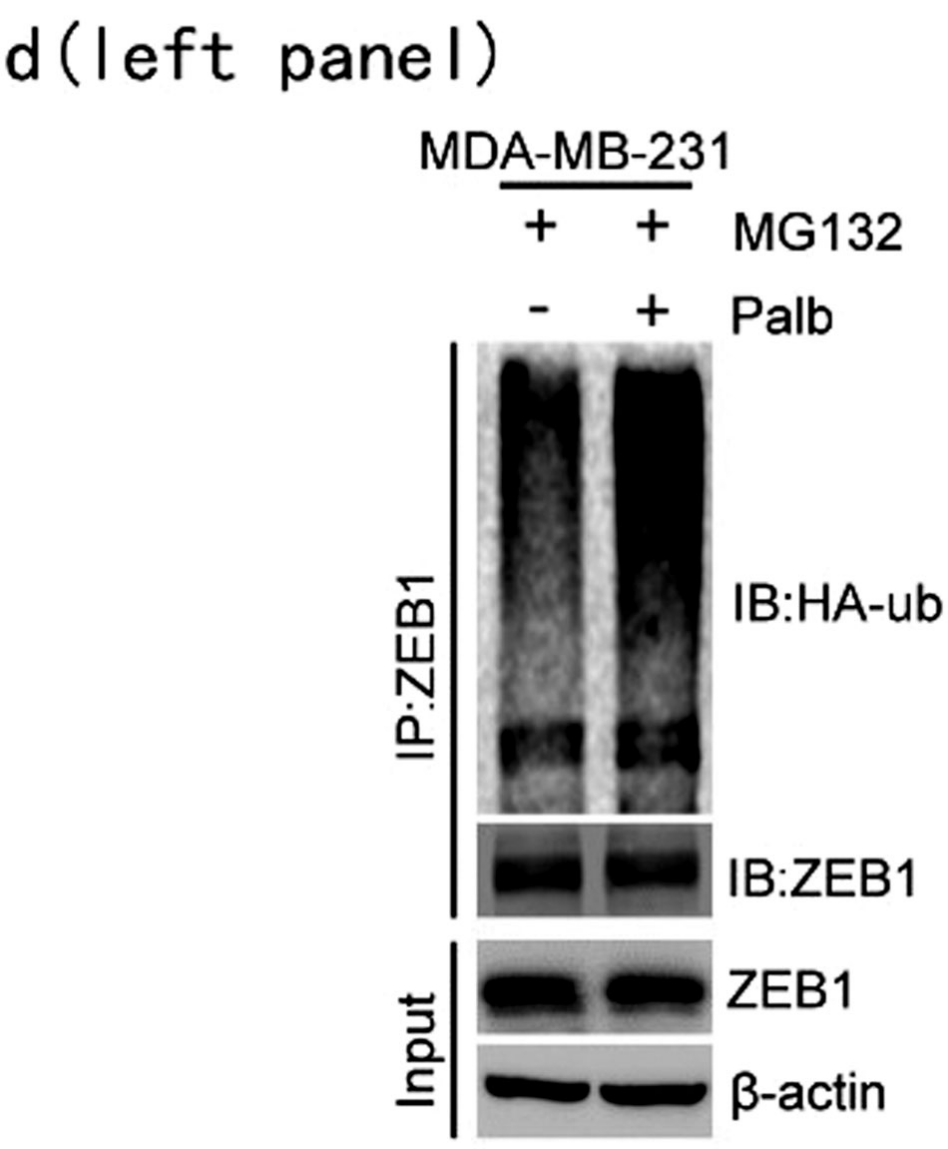


Figure. 1d (left panel). Coimmunoprecipitation analysis of ZEB1 protein ubiquitination in MDA-MB-231 cells after treatment with palbociclib for 48 h. The cells were treated with MG132 for 12 h prior to harvest.

The authors noticed some inaccurate information in the “Tissue microarray and immunohistochemistry (IHC) scoring” section of the materials & methods that needs to be corrected. The sentence of “Twenty fresh breast invasive ductal carcinoma tissues were obtained from the First Affiliated Hospital of Chongqing Medical University (Chongqing, China), 100 breast invasive ductal carcinoma tissues were obtained from Alenabio Biotechnology Ltd., Xi’an, China (catalog number: BC081120c), and 145 breast invasive ductal carcinoma tissues with overall survival rate were obtained from Shanghai Outdo Biotech Co., Ltd., China. All of the patients had histologically confirmed invasive ductal carcinoma breast cancer (Tables S4–S7).” is changed to “Twenty fresh breast carcinoma tissues were obtained from the First Affiliated Hospital of Chongqing Medical University (Chongqing, China) and the study was approved by the Ethics Committee of the First Affiliated Hospital of Chongqing Medical University, tissue microarrays containing 100 breast carcinoma tissues were obtained from Alenabio Biotechnology Co., Ltd., Xi’an, China (catalog number: BC081120c), and tissue microarrays containing 145 breast carcinoma tissues with overall survival rate were obtained from Shanghai Outdo Biotech Co., Ltd., China. Written informed consent for using clinical information and tissue samples was obtained from all patients. All of the patients had histologically confirmed breast carcinoma (Tables S4–S7)”. The key findings of the article are not affected by these corrections.

The original article has been corrected.

## Supplementary information

Fig S5d

