# Supplementary material for: CDK4/6 inhibition blocks cancer metastasis through a USP51-ZEB1-dependent deubiquitination mechanism
Source: Signal Transduct Target Ther. 2020 Mar 11;5:25. doi: 10.1038/s41392-020-0118-x (PMC7064488; doi:10.1038/s41392-020-0118-x)
Supplement: Supplementary file 1 — Supplementary information [file 41392_2020_118_MOESM1_ESM.docx]

Supplementary Materials for

CDK4/6 inhibition blocks cancer metastasis through a USP51-ZEB1-dependent deubiquitination mechanism

Zhen Zhang^1^, Jianjun Li^1^, Yang Ou^1^, Guang Yang^2^, Kaiyuan Deng^1^, Qiong Wang^1^, Zhaoyang Wang^1^, Wenhao Wang^1^, Quansheng Zhang^3^, Hang Wang^1^, Wei Sun^1^, Peiqing Sun^4^, Shuang Yang^1*^

^1^Tianjin Key Laboratory of Tumor Microenvironment and Neurovascular Regulation, Medical College of Nankai University, Tianjin 300071, China

^2^College of Pharmacy, Nankai University, Tianjin 300071, China

^3^Tianjin Key Laboratory of Organ Transplantation, Tianjin First Center Hospital, Tianjin 300192, China

^4^Department of Cancer Biology, Wake Forest University School of Medicine, Winston-Salem, NC 27157, USA

*Corresponding author: Shuang Yang at Medical College of Nankai University; 94 Weijin Road, Tianjin 300071, China; Tel: +86-22-23509557; Fax: +86-22-23505501;

E-mail: [yangshuang@nankai.edu.cn](mailto:yangshuang@nankai.edu.cn)

**This file includes:** Figures S1 to S7, Tables S1 to S7.

Figure. S1.


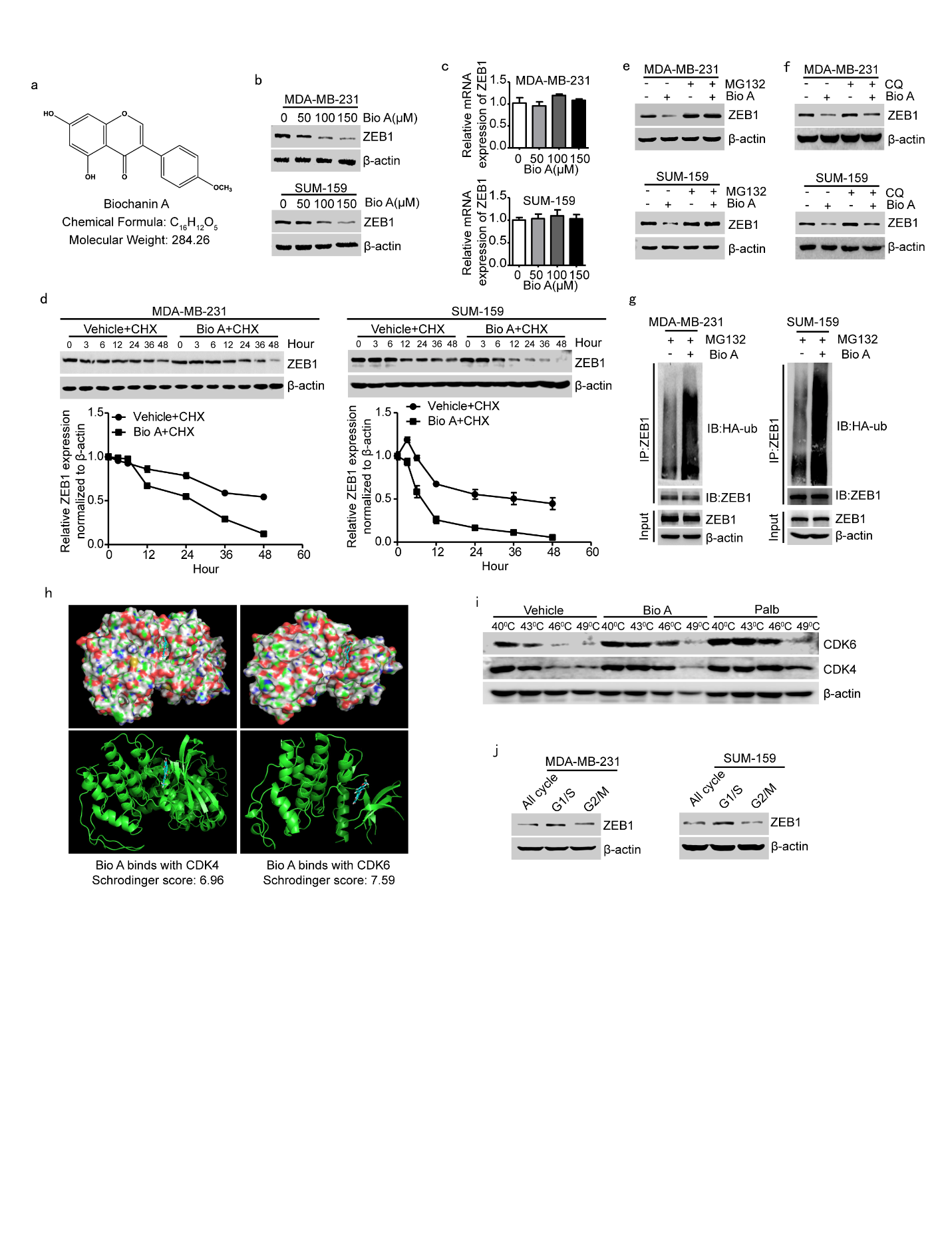
 **Figure. S1. Biochanin A induces ZEB1 protein degradation in breast cancer cells. a** Structure of Biochanin A. **b, c** Western blotting **(b)** and Q-PCR **(c)** analysis of ZEB1 expression in MDA-MB-231 and SUM-159 cells by treatment with the indicated concentrations of Biochanin A for 48 h. **d** CHX pulse-chase analysis of ZEB1 protein stability in MDA-MB-231 and SUM-159 cells by treatment with Biochanin A at the indicated time points. The results were normalized to the levels of β-actin. **e**, **f** Western blottingof ZEB1 protein expression in MDA-MB-231 and SUM-159 cells by treatment with MG132 **(e)** and CQ **(f)** in the presence or absence of Biochanin A. **g** Co-immunoprecipitation analysis of ZEB1 protein ubiquitination in MDA-MB-231 and SUM-159 cells by treatment with Biochanin A for 48 h. Cells were treated with MG132 for 12 h prior to harvest. **h** The binding mode of Biochanin A with CDK4/6. **i** Cellularthermal shift assay of the binding of CDK4/6 with Biochanin A or Palbciclib in MDA-MB-231 cells. **j** MDA-MB-231 and SUM-159 cells were synchronized in the G1/S phase using methotrexate and in the G2/M phase using colchicine, respectively. Western blotting analysis of ZEB1 protein expression were performed. β-actin was used as a loading control.

**Figure. S2.** **
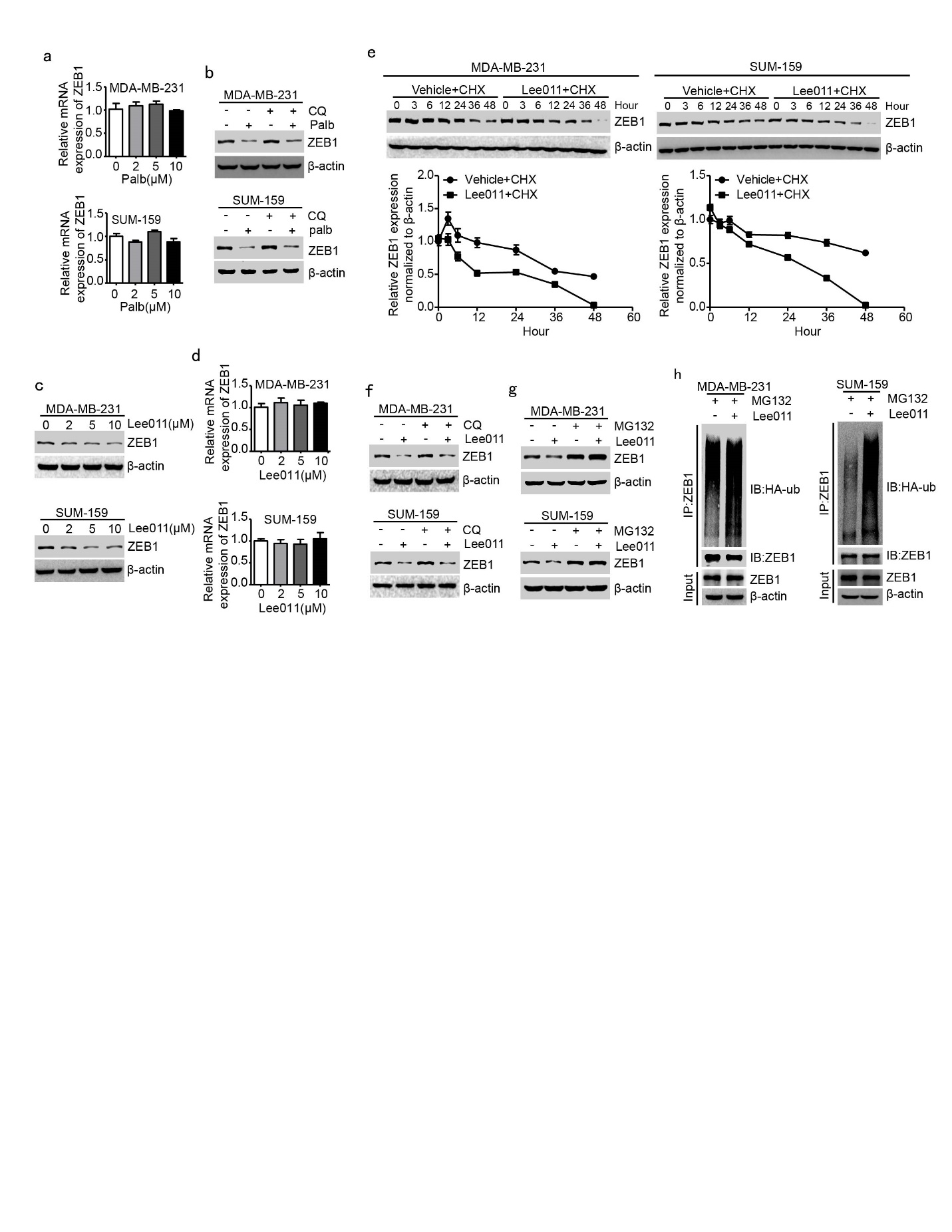
**

**Figure. S2. CDK4/6 Inhibition induces ZEB1 protein degradation. a** Q-PCR of ZEB1 expression in MDA-MB-231 and SUM-159 cells by treatment with the indicated concentrations of Palbociclib for 48 h. **b** Western blotting of ZEB1 protein expression in MDA-MB-231 and

SUM-159 cells by treatment with CQ in the presence or absence of Palbociclib. **c, d** Western blotting **(c)** and Q-PCR **(d)** analysis of ZEB1 expression in MDA-MB-231 and SUM-159 cells by treatment with the indicated concentrations of Lee011 for 48 h. **e** CHX pulse-chase analysis of ZEB1 protein stability in MDA-MB-231 and SUM-159 cells by treatment with Lee011 at the indicated time points. The results were normalized to the levels of β-actin. **f, g** Western blotting of ZEB1 protein expression in MDA-MB-231 and SUM-159 cells by treatment with CQ **(f)** and MG132 **(g)** in the presence or absence of Lee011. **h** Co-immunoprecipitation analysis of ZEB1 protein ubiquitination in MDA-MB-231 and SUM-159 cells by treatment with Lee011 for 48 h. The cells were treated with MG132 for 12 h prior to harvest.

Figure. S3.


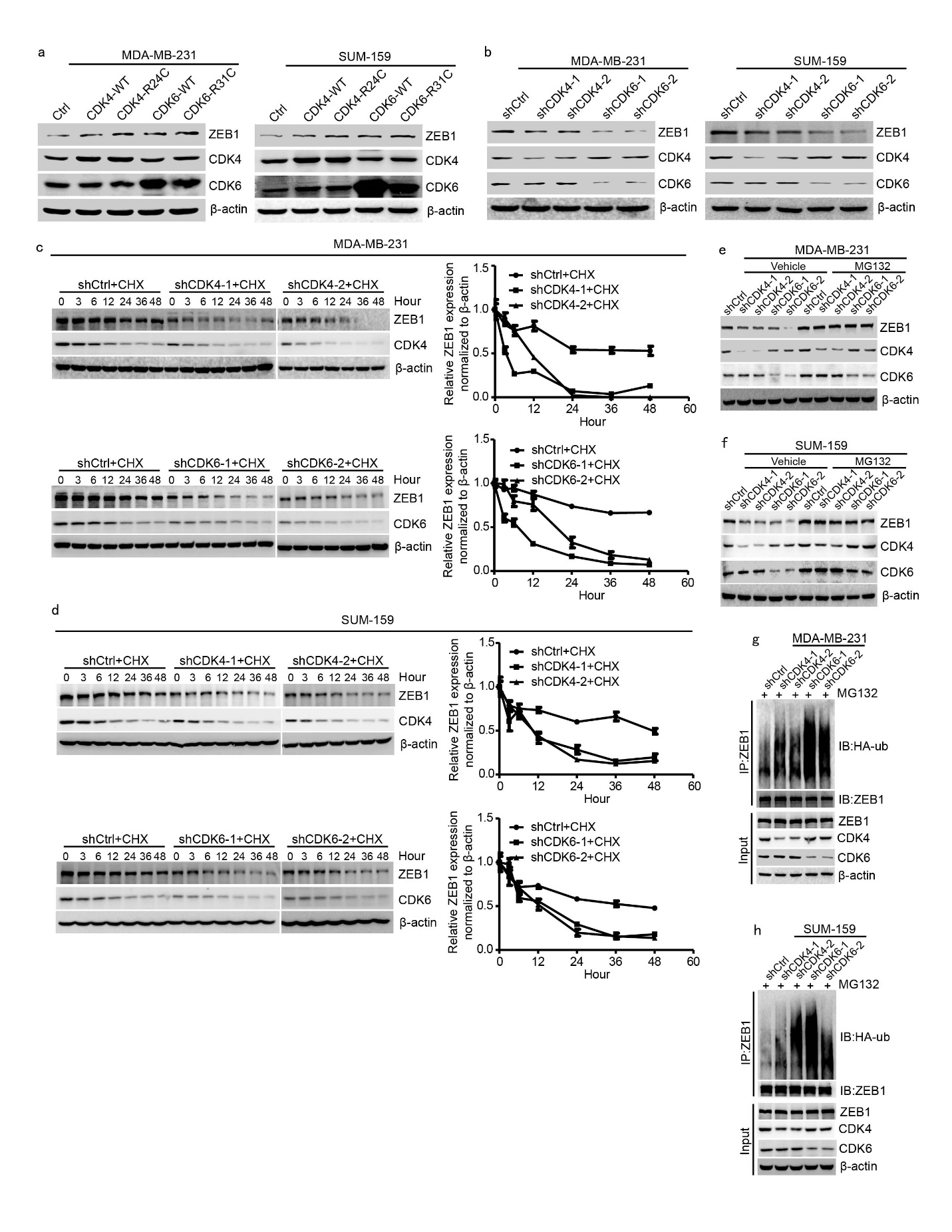
**Figure. S3. CDK4/6 Inhibition induces ZEB1 protein degradation. a** Western blotting of ZEB1 protein expression in MDA-MB-231 and SUM-159 cells that were overexpressed with wild-type or hyperactive mutant forms (CDK4-R24C and CDK4-R31C) of CDK4/6. **b** Western blotting of ZEB1 protein expression in CDK4/6-interfered MDA-MB-231 and SUM-159 cells. **c, d** CHX pulse-chase analysis of ZEB1 protein stability in CDK4/6-interfered MDA-MB-231 **(c)** and SUM-159 **(d)** cells at the indicated time points. The results were normalized to the levels of β-actin. **e, f** Western blotting of ZEB1 protein expression in CDK4/6-interfered MDA-MB-231 **(e)** and SUM-159 **(f)** cells by treatment with MG132. **g, h** Co-immunoprecipitation analysis of ZEB1 protein ubquitination in CDK4/6-interfered MDA-MB-231 **(g)** and SUM-159 **(h)** cells. The cells were treated with MG132 for 12 h prior to harvest.

Figure. S4.


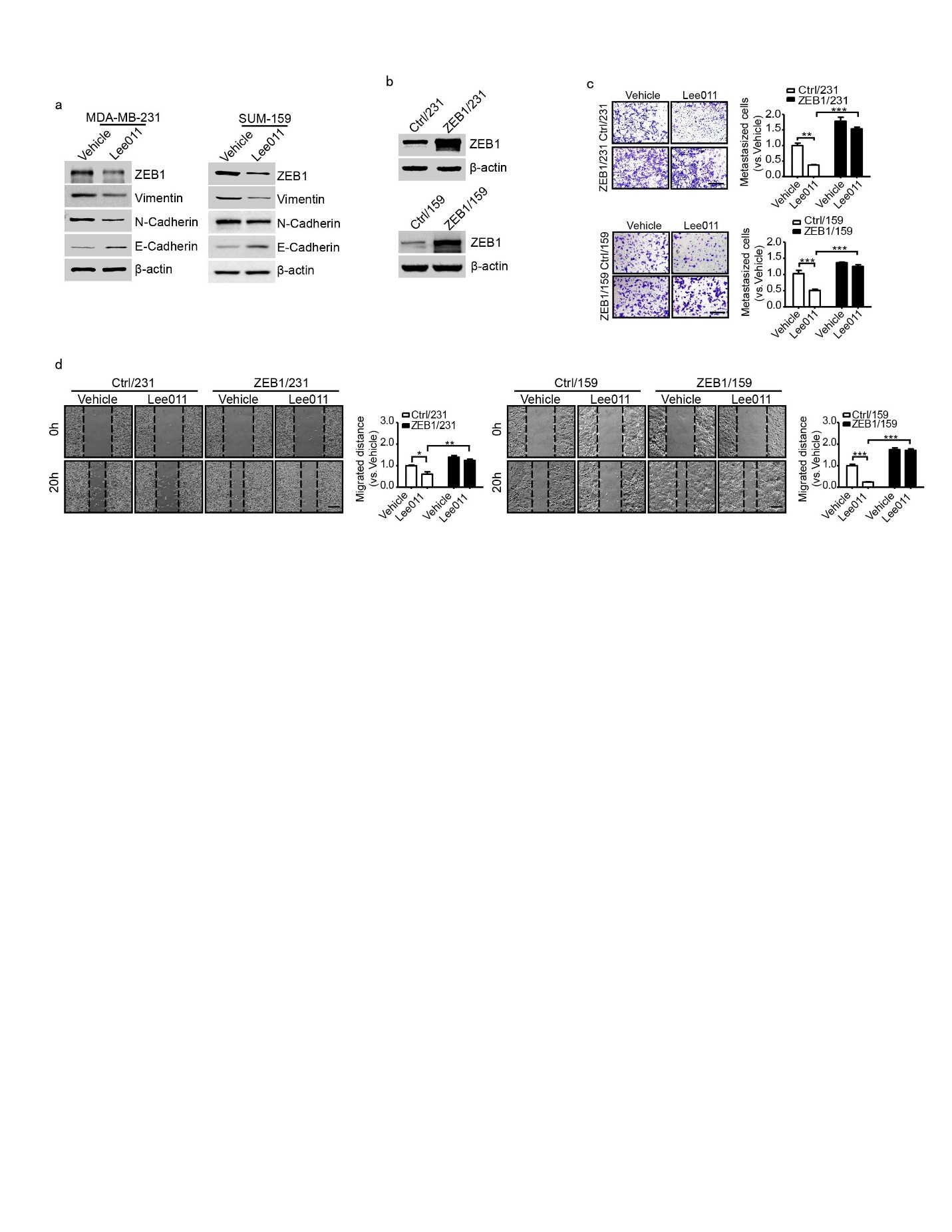


**Figure. S4. CDK4/6 inhibition with Lee011 inhibits breast cancer cell migration via regulating ZEB1.** **a** Western blotting of the EMT markers in MDA-MB-231 and SUM-159 cells by treatment with Lee011 for 48 h. **b** Western blotting of ZEB1 protein expression in MDA-MB-231 and SUM-159 cells that were overexpressed with ZEB1. **c, d** Transwell migration **(c)** and wound-healing **(d)** analysis in ZEB1-expressing MDA-MB-231 and SUM-159 cells by treatment with Lee011. Scale bars, 100 μm. **P* < 0.05, ***P* < 0.01, ****P* < 0.001 vs. respective control by unpaired Student’s t-test.

Figure. S5.


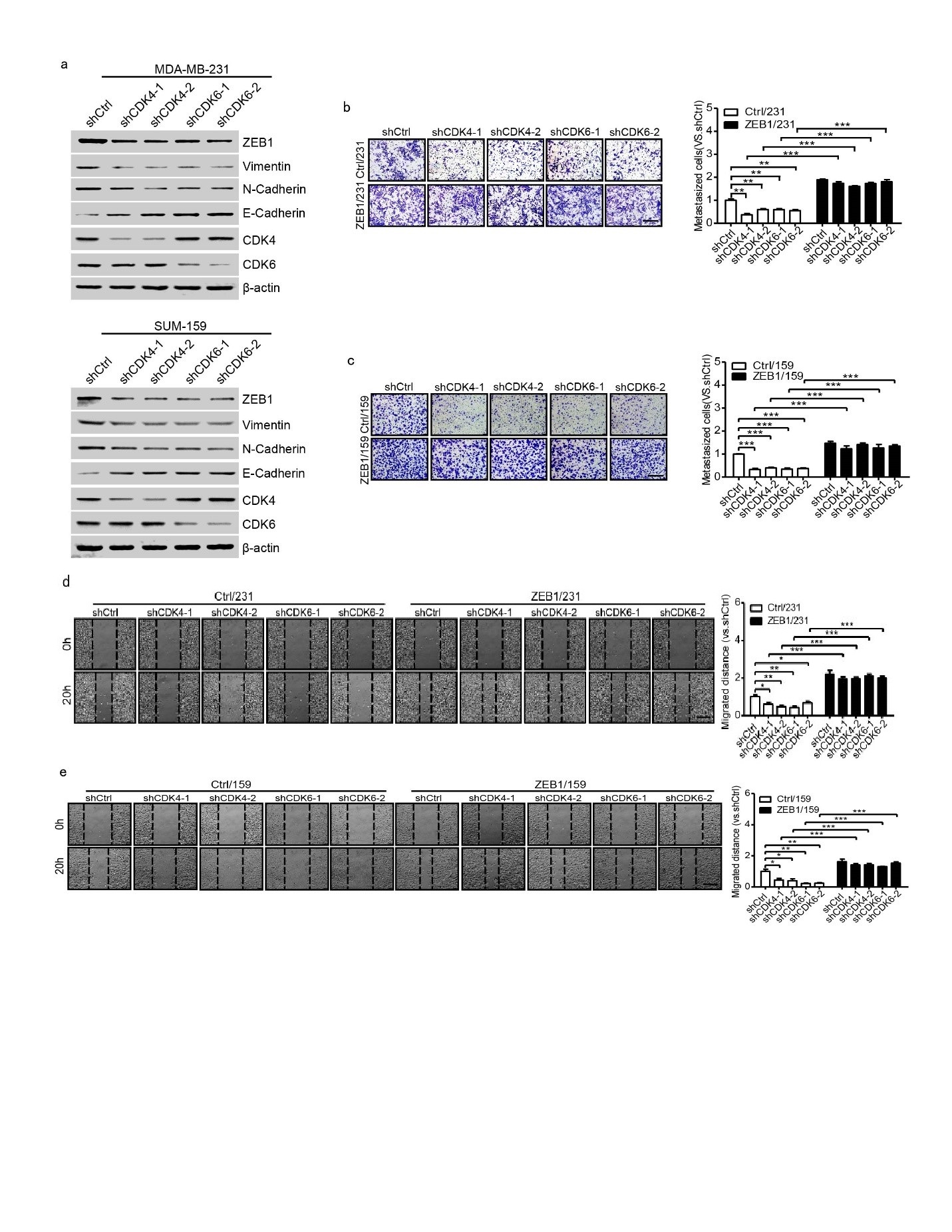
`

**Figure. S5. CDK4/6 knockdown inhibits breast cancer cell migration via regulating ZEB1. a** Western blotting of the EMT markers in CDK4/6-interfered MDA-MB-231 and SUM-159 cells. **b, c** Transwell migration analysis in CDK4/6-interfered MDA-MB-231 **(b)** and SUM-159 **(c)** cells in the presence or absence of rescued ZEB1 expression. Scale bars, 100 μm. ***P* < 0.01, ****P* < 0.001 vs respective control by unpaired Student’s t-test. **d, e** Wound healing analysis in CDK4/6-interfered MDA-MB-231 **(d)** and SUM-159 **(e)** cells in the presence or absence of rescued ZEB1 expression. Scale bars, 100 μm. **P* < 0.05, ***P* < 0.01, ****P* < 0.001 vs. respective control by unpaired Student’s t-test.

Figure. S6.


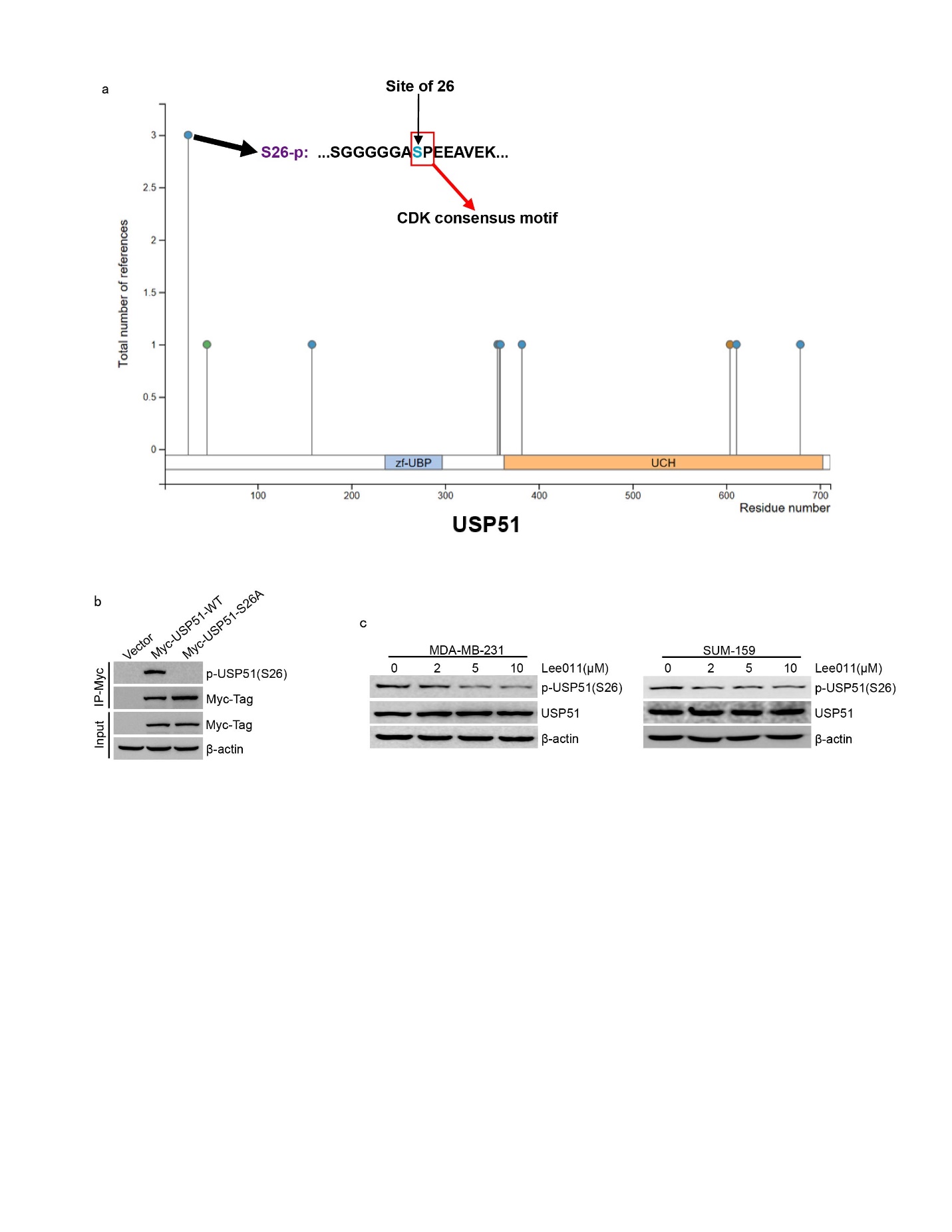


**Figure. S6. CDK4/6 phosphorylates USP51 at Ser26. a** The phosphorylation site of USP51 identified by PhosphoSitePlus® database. S26-p means that Ser26 is a major phosphorylation site on USP51. **b** Myc-USP51-WT or Myc-USP51-S26A was transfected in 293T cells. Cell lysates were subjected to co-immunoprecipitation with anti-Myc antibody and the phosphorylation of Ser26 on USP51 was examined. **c** Western blotting of S26-USP51 phosphorylation in MDA-MB-231 and SUM-159 cells by treatment with the indicated concentrations of Lee011 for 48 h.

Figure. S7.


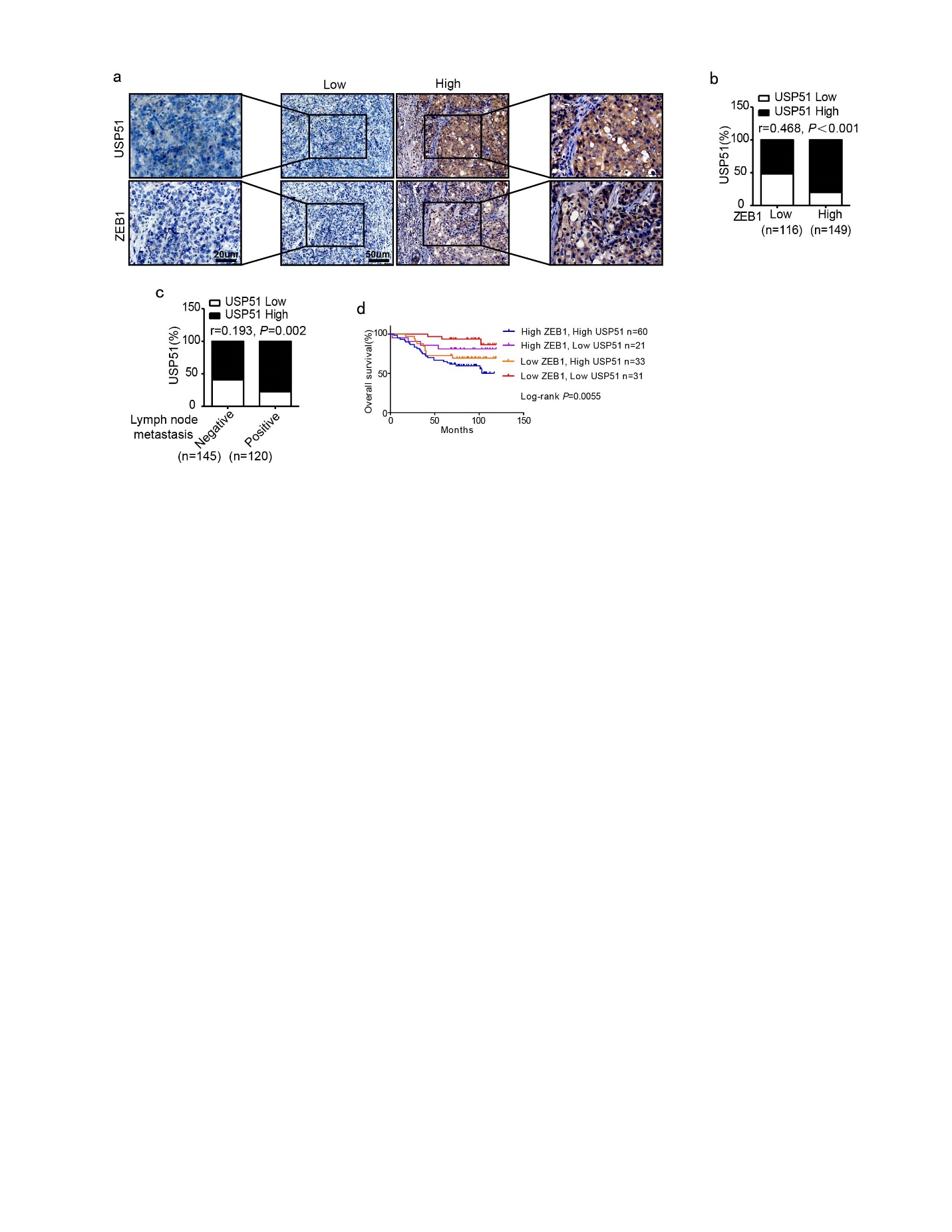


**Figure. S7. The expression of USP51 and ZEB1 is positively correlated in human breast cancer. a** Representative images of the immunohistochemical staining of USP51 and ZEB1 in serial sections of the same tumor from two cases. Scale bars, 20 μm and 50 μm. **b** A positive correlation between the expression of USP51 and ZEB1 in the 265 human breast cancer samples.

r = 0.468, *P* < 0.001 by Spearman’s rank correction test. **c** Increased expression of UPS51 in metastatic breast cancer. r = 0.193, *P* = 0.002 by chi-square test. **d** Kaplan–Meier curves showing shorter overall survival of patients with concomitantly high expression of UPS51 and ZEB1 in their breast tumors. *P* = 0.0055 by log-rank test.

Table S1. Natural compounds library.

| No. | Name | ZEB1/β-actin expression  normalized to Vehicle（$\bar{\boldsymbol{X}}$±S） |
| --- | --- | --- |
| 1 | Aescine A | 0.81 ± 0.079 |
| 2 | Aescine B | 0.77 ± 0.080 |
| 3 | Aescine C | 1.04 ± 0.040 |
| 4 | Aescine D | 1.04 ± 0.039 |
| 5 | Amentoflavone | 0.93 ± 0.079 |
| 6 | Atractylenolide I | 1.00 ± 0.081 |
| *7* | ***Biochanin A*** | ***0.44 ± 0.095*** |
| 8 | Borneol | 1.10 ± 0.021 |
| 9 | Casticin | 1.09 ± 0.097 |
| 10 | Catharanthine | 1.02 ± 0.134 |
| 11 | Chlorogenic acid | 1.10 ± 0.096 |
| 12 | Corilagin | 1.14 ± 0.073 |
| 13 | Corosolic acid | 1.07 ± 0.043 |
| 14 | Curcumol | 1.11 ± 0.063 |
| 15 | Epimedin A | 1.16 ± 0.085 |
| 16 | Epimedin B | 1.18 ± 0.059 |
| 17 | Epimedin C | 1.09 ± 0.074 |
| 18 | Geniposide | 1.04 ± 0.023 |
| 19 | Ginsenoside Ro | 1.00 ± 0.204 |
| 20 | Isomangiferin | 1.00 ± 0.022 |
| 21 | Jujuboside B | 0.97 ± 0.041 |
| 22 | Lathyrol | 1.11 ± 0.058 |
| 23 | Limonin | 1.03 ± 0.098 |
| 24 | Mangostin | 0.97 ± 0.142 |
| 25 | Mogroside IV | 1.05 ± 0.136 |
| 26 | Mogroside VI | 0.99 ± 0.188 |
| 27 | Phellodendrine chloride | 0.98 ± 0.168 |
| 28 | Praeruptorin C | 1.07 ± 0.073 |
| 29 | Pseudolaric acid A-O-β-D-glucopyranoside | 1.03 ± 0.19 |
| 30 | Quercetin-3-rhamnoside | 1.01 ± 0.007 |
| 31 | Rosin | 1.00 ± 0.085 |
| 32 | Saikosaponin A | 1.03 ± 0.066 |
| 33 | Scutellarin | 1.14 ± 0.065 |
| 34 | Secoisolariciresinol diglucoside | 1.17 ± 0.059 |
| 35 | Sennoside A | 1.10 ± 0.067 |
| 36 | Sennoside C | 1.06 ± 0.102 |
| 37 | Siamenoside I | 1.01 ± 0.070 |
| 38 | Tectoridin | 1.06 ± 0.106 |
| 39 | Tiliroside | 1.06 ± 0.129 |
| 40 | Yohimbine hydrochloride | 1.02 ± 0.021 |
| 41 | 2-Hydroxyadenosine | 1.01 ± 0.138 |
| 42 | 20(S)-Ginsenoside Rh2 | 1.05 ± 0.024 |

Table S2. Primer used in this study.

| Construction of ZEB1 expression vector | |
| --- | --- |
| ZEB1 forward | 5’-ATGGCGGATGGCCCCAGG‑3’ |
| ZEB1 reverse | 5’- TTAGGCTTCATTTGTCTTTTC‑3’ |
| ZEB1-NZF forward | 5’-ATGGCGGATGGCCCCAGG‑3’ |
| ZEB1-NZF reverse | 5’-CTAGTTAACAGAAAGTTGTTC‑3’ |
| ZEB1-HD forward | 5’-ATGAACCAAATTAAAACTGAA‑3’ |
| ZEB1-HD reverse | 5’-CTATGCACCCTCAGCTGTGTA‑3’ |
| ZEB1-CZF forward | 5’-ATGGCACAAGAAGAGCCACAA‑3’ |
| ZEB1-CZF reverse | 5’- TTAGGCTTCATTTGTCTTTTC‑3’ |
| Construction of CDK4 expression vector | |
| CDK4 forward | 5’-ATGGCTACCTCTCGATATGAG‑3’ |
| CDK4 reverse | 5’-TCACTCCGGATTACCTTCATC‑3’ |
| Construction of CDK6 expression vector | |
| CDK6 forward | 5’-ATGGAGAAGGACGGCCTG‑3’ |
| CDK6 reverse | 5’-TCAGGCTGTATTCAGCTCCGA‑3’ |
| Construction of shRNA vector | |
| shCDK4-1 | 5’-GAGATTACTTTGCTGCCTTAA‑3’ |
| shCDK4-2 | 5’-GCAGAGATGTTTCGTCGAAAG‑3’ |
| shCDK6-1 | 5’-CAGATGTTGATCAACTAGGAA‑3 |
| shCDK6-2 | 5’-GGATATGATGTTTCAGCTTCT‑3’ |
| shUSP51-1 | 5’-GCATCTGATATGGATCCATGC‑3’ |
| shUSP51-2 | 5’-GCTACCAGGAGTCTACTAAAC‑3’ |
| Quantitative RT-PCR | |
| human ZEB1 forward | 5’-CAGCTTGATACCTGTGAATGGG-3’ |
| human ZEB1 reverse | 5’-TATCTGTGGTCGTGTGGGACT-3’ |
| human GAPDH forward | 5’-GGAGCGAGATCCCTCCAAAAT-3’ |
| human GAPDH reverse | 5’-GGCTGTTGTCATACTTCTCATGG-3’ |

Table S3. Antibodies.

| Antibodies | Species | Application | Manufacturer | Catalog No. | Dilution |
| --- | --- | --- | --- | --- | --- |
| anti-ZEB1 | Rabbit | IHC | Abcam | ab87280 | 1:100 |
|  | Rabbit | IB | Santa Cruz | sc-25388 | 1:1000 |
|  | Rabbit | IP | Proteintech | 21544-1-AP | 4μg/ml |
| anti-Flag-Tag | Mouse | IP | Sigma-Aldrich | F1804 | 3μg/ml |
|  | Rabbit | IB | CST | 2368S | 1:1000 |
| anti-β-actin | Mouse | IB | Santa Cruz | sc-47778 | 1:1000 |
| anti-HA-Tag | Rabbit | IB | CST | 3724S | 1:1000 |
| anti-CDK4 | Rabbit | IB | Proteintech | 11026-1-AP | 1:1000 |
| anti-CDK6 | Rabbit | IB | Proteintech | 14052-1-AP | 1:1000 |
| anti-Vimentin | Rabbit | IB | CST | 5741S | 1:1000 |
| anti-N-Cadherin | Mouse | IB | BD Biosciences | 610920 | 1:1000 |
| anti-E-Cadherin | Mouse | IB | CST | 14472S | 1:1000 |
| anti-USP51 | Rabbit | IB | Sigma-Aldrich | SAB1305451 | 1:1000 |
|  |  | IP |  |  | 5μg/ml |
|  |  | IHC |  |  | 1:200 |
| anti-Myc-Tag | Mouse | IP | CST | 2276S | 3μg/ml |
|  | Rabbit | IB | CST | 2278S | 1:1000 |
| anti- *p*-serine | Rabbit | IB | Abcam | ab9332 | 1:1000 |
| anti-p-USP51(S26) | Rabbit | IB | Abclonal | Custom order | 1:1000 |
|  |  | IHC |  |  | 1:200 |
| anti-*p*-RB | Rabbit | IHC | CST | 8516S | 1:200 |
|  |  | IB |  |  | 1:1000 |

IHC: Immunohistochemistry; IB: Immunoblotting; IP: Immunoprecipitation.

Table S4. *p*-RB expression and clinicopathologic features of breast tumors.

| **Clinicopathologic features** | **Number (n=265)** | ***p*-RB expression** | | ***P* value** |
| --- | --- | --- | --- | --- |
|  |  | **High** | **Low** |  |
| **Age (years)** |  |  |  | *P* = 0.889 |
| ≤40 | 30 | 13 | 17 |  |
| ＞40 | 235 | 105 | 130 |  |
| **Tumor size (cm)** |  |  |  | *P* = 0.034 |
| ≤2.0 | 52 | 15 | 37 |  |
| ＞2.0, ≤5.0 | 167 | 79 | 88 |  |
| ＞5.0 | 46 | 24 | 22 |  |
| **Lymph node metastasis** |  |  |  | *P* = 0.009 |
| Positive | 120 | 64 | 56 |  |
| Negative | 145 | 54 | 91 |  |

Table S5. *p*-USP51 expression and clinicopathologic features of breast tumors.

| **Clinicopathologic features** | **Number (n=265)** | ***p*-USP51 expression** | | ***P* value** |
| --- | --- | --- | --- | --- |
|  |  | **High** | **Low** |  |
| **Age (years)** |  |  |  | *P* = 0.948 |
| ≤40 | 30 | 22 | 8 |  |
| ＞40 | 235 | 171 | 64 |  |
| **Tumor size (cm)** |  |  |  | *P* = 0.278 |
| ≤2.0 | 52 | 36 | 16 |  |
| ＞2.0, ≤5.0 | 167 | 127 | 40 |  |
| ＞5.0 | 46 | 30 | 16 |  |
| **Lymph node metastasis** |  |  |  | *P* ＜ 0.001 |
| Positive | 120 | 102 | 18 |  |
| Negative | 145 | 91 | 54 |  |

Table S6. ZEB1 expression and clinicopathologic features of breast tumors.

| **Clinicopathologic features** | **Number (n=265)** | **ZEB1 expression** | | ***P* value** |
| --- | --- | --- | --- | --- |
|  |  | **High** | **Low** |  |
| **Age (years)** |  |  |  | *P* = 0.959 |
| ≤40 | 30 | 17 | 13 |  |
| ＞40 | 235 | 132 | 103 |  |
| **Tumor size (cm)** |  |  |  | *P* = 0.210 |
| ≤2.0 | 52 | 24 | 28 |  |
| ＞2.0, ≤5.0 | 167 | 96 | 71 |  |
| ＞5.0 | 46 | 29 | 17 |  |
| **Lymph node metastasis** |  |  |  | *P* ＜ 0.001 |
| Positive | 120 | 82 | 38 |  |
| Negative | 145 | 67 | 78 |  |

Table S7. USP51 expression and clinicopathologic features of breast tumors.

| **Clinicopathologic features** | **Number (n=265)** | **USP51 expression** | | ***P* value** |
| --- | --- | --- | --- | --- |
|  |  | **High** | **Low** |  |
| **Age (years)** |  |  |  | *P* = 0.601 |
| ≤40 | 30 | 19 | 11 |  |
| ＞40 | 235 | 160 | 75 |  |
| **Tumor size (cm)** |  |  |  | *P* = 0.126 |
| ≤2.0 | 52 | 30 | 22 |  |
| ＞2.0, ≤5.0 | 167 | 120 | 47 |  |
| ＞5.0 | 46 | 29 | 17 |  |
| **Lymph node metastasis** |  |  |  | *P* = 0.002 |
| Positive | 120 | 93 | 27 |  |
| Negative | 145 | 86 | 59 |  |
