# Supplementary figures and images for: Correction: CDK4/6 inhibition blocks cancer metastasis through a USP51-ZEB1-dependent deubiquitination mechanism
Source: Signal Transduct Target Ther. 2024 Oct 4;9:265. doi: 10.1038/s41392-024-01972-4 (PMC11452405; doi:10.1038/s41392-024-01972-4)

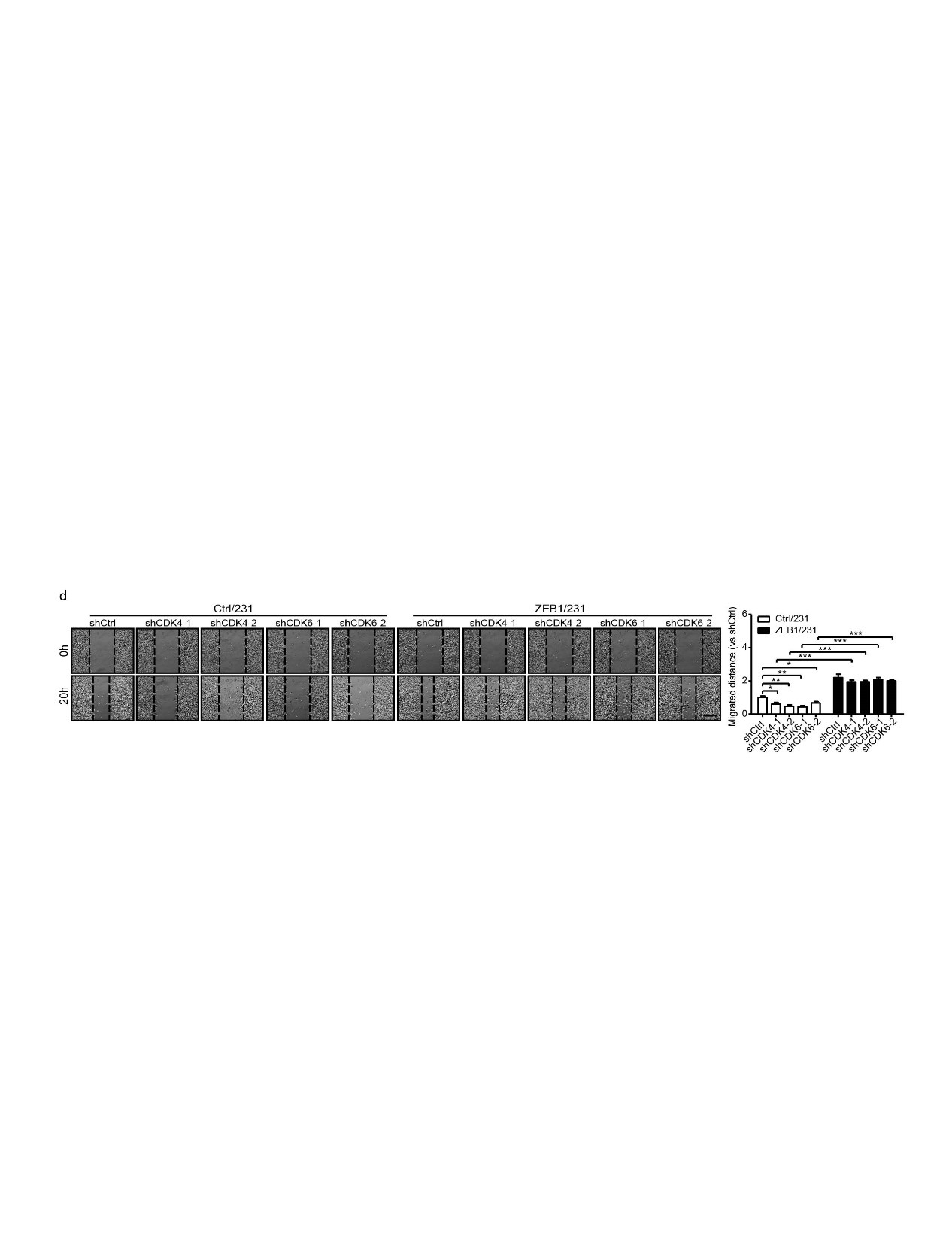

Supplement: Supplementary file 1 — Fig S5d [file 41392_2024_1972_MOESM1_ESM.jpg]
